# Inferior Frontal Gyrus Alterations in Freezing of Gait in Parkinson's Disease Without Evidence for Overall Hemispheric Lateralization: A Systematic Review and Activation Likelihood Estimation Meta‐Analysis

**DOI:** 10.1111/ejn.70739

**Published:** 2026-10-29

**Authors:** Seira Taniguchi, Hannah Deckmyn, Moran Gilat

**Affiliations:** ^1^ Department of Rehabilitation Sciences, Neurorehabilitation Research Group (eNRGy) KU Leuven Leuven Belgium; ^2^ Department of Neurology The University of Osaka Graduate School of Medicine Suita Japan

**Keywords:** activation likelihood estimation, coordinate‐based meta‐analysis, freezing of gait, neuroimaging, Parkinson's disease

## Abstract

Freezing of gait (FOG) is one of the most debilitating motor symptoms of Parkinson's disease (PD). Prior studies have suggested a potential right‐hemispheric preference in the pathophysiology of FOG. Yet, there is limited evidence regarding which brain regions are most consistently associated with FOG and whether these alterations indeed show right‐hemispheric lateralization. We aimed to identify brain regions associated with FOG based on multimodal neuroimaging findings and to examine their hemispheric lateralization. A systematic review across multiple neuroimaging modalities was performed, followed by an activation likelihood estimation (ALE) coordinate‐based meta‐analysis to assess convergent brain alterations in PD patients with FOG (freezers) versus those without FOG (nonfreezers). Hemispheric lateralization was assessed using the distribution of reported stereotactic coordinates. The systematic review included 108 articles, of which 33 were included in the primary ALE meta‐analysis. We identified a significant convergent cluster in the right inferior frontal gyrus (IFG) pars opercularis/dorsolateral prefrontal cortex in PD freezers compared with nonfreezers. No evidence of overall hemispheric lateralization was observed based on the distribution of reported coordinates. Our findings identify the right IFG as a consistent neural correlate of FOG, potentially reflecting heightened frontal recruitment, with no clear evidence of hemispheric lateralization. The lack of further consistency in brain regions could reflect the known heterogeneity in the pathophysiology of FOG.

## Graphical abstract

Our systematic review included 108 neuroimaging articles examining freezing of gait in Parkinson's disease, with 33 included in the primary activation likelihood estimation meta‐analysis. We assessed spatial convergence and hemispheric lateralization of reported abnormalities, identifying the right inferior frontal gyrus as the main convergent region, with no evidence of overall hemispheric lateralization.

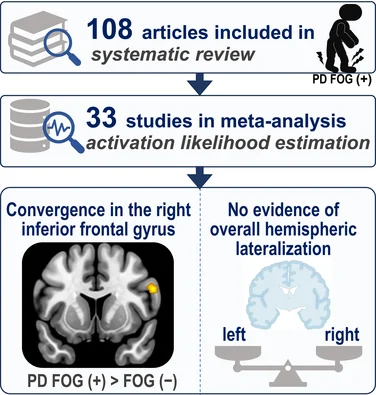

**Abbreviations**
ALE: activation likelihood estimation
CLR: cerebellar locomotor region
DLPFC: dorsolateral prefrontal cortex
fMRI: functional magnetic resonance imaging
FOG: freezing of gait
FWE: family‐wise error
IFG: inferior frontal gyrus
MLR: mesencephalic locomotor region
MNI: Montreal Neurological Institute
MRI: magnetic resonance imaging
PD: Parkinson's disease
PET: positron emission tomography
PRISMA: Preferred Reporting Items for Systematic Reviews and Meta‐Analyses
ROI: region of interest
SMA: supplementary motor area
SPECT: single‐photon emission computed tomography

## 1 Introduction

Freezing of gait (FOG) is one of the most disabling motor symptoms in people with Parkinson's disease (PD) (Giladi et al. 1997), yet its underlying pathophysiology remains incompletely understood. Beyond the fundamental PD pathology—characterized by a reduction in striatal dopamine levels due to the loss of dopaminergic neurons in the substantia nigra pars compacta—several reviews summarize the regional and circuit alterations in PD patients with FOG (i.e., freezers) compared to those without FOG (i.e., nonfreezers). Using different imaging modalities (e.g., magnetic resonance imaging [MRI], positron emission tomography [PET], and single‐photon emission computed tomography [SPECT]), these studies highlighted widespread functional and structural abnormalities across both cortical and subcortical regions (Snijders et al. 2016; Bardakan et al. 2022; Weiss et al. 2020). A review by Bardakan et al. highlighted that resting‐state fMRI studies mainly reveal cortico‐striatal‐brainstem abnormalities (e.g., supplementary motor area [SMA], striatum, and mesencephalic locomotor region [MLR]) and impaired dorsal visuomotor processing, while task‐based fMRI studies demonstrate a disruption in cortico‐subcortical connectivity and an increased recruitment of parieto‐occipital regions (Bardakan et al. 2022). A previous coordinate‐based meta‐analysis (i.e., activation likelihood estimation [ALE] analysis) by Gilat et al. revealed that the cerebellar locomotor region (CLR) showed the most consistent gait‐related activation in PD versus age‐matched healthy adults. While that prior review provides an understanding of the functional neural signatures of Parkinsonian gait during gait‐related neuroimaging tasks (Gilat et al. 2019), it neither explicitly focused on freezers versus nonfreezers nor addressed structural alterations that have also been identified in PD freezers (D'Cruz et al. 2021; Fling et al. 2014; Fling et al. 2013). Consequently, there is still no convincing evidence regarding exactly “*where*” the most consistent neural alterations occur in freezers compared with nonfreezers. Specifically, to date, whether left‐ or right‐hemispheric alterations are more dominant (i.e., hemispheric lateralization) in FOG remains controversial. Clarifying this issue may provide additional insight into whether FOG‐related neural alterations preferentially involve one hemisphere or are more broadly bilateral. This could help inform future therapeutic strategies, such as neuromodulation techniques. Some neuroimaging studies have suggested a possible right‐hemispheric vulnerability in FOG (Fling et al. 2013; Craig et al. 2026). For example, Fling et al. reported reduced structural connectivity of the pedunculopontine nucleus with the cerebellum, thalamus, and frontal/prefrontal cortices specifically in the right hemisphere of freezers (Fling et al. 2013). Craig et al. extended these findings by showing right‐asymmetric atrophy of the nucleus basalis of Meynert (Craig et al. 2026). However, this right‐hemispheric hypothesis has since been questioned (Hall et al. 2015). Conversely, Rubino et al. localized parietal gray‐matter atrophy in the left posterior parietal cortex in freezers (Rubino et al. 2014), while our previous study showed effective connectivity from the left dorsolateral prefrontal cortex to the bilateral MLR in freezers (Taniguchi et al. 2024). These inconsistencies highlight the need for a systematic evaluation of the brain regions most consistently associated with FOG, as well as their hemispheric lateralization. Here, we therefore systematically reviewed and performed an ALE meta‐analysis to reveal which brain regions have been most consistently associated with FOG in the literature. Specifically, the current study's primary aims were to (1) systematically summarize FOG‐specific findings of available neuroimaging experiments using various modalities (e.g., resting‐state/task‐based fMRI, structural MRI, PET/SPECT studies) and subsequently (2) identify spatial convergence across the results of the available neuroimaging studies on PD patients with and without FOG. The secondary objective was to evaluate whether FOG‐related neuroimaging findings show right‐hemispheric predominance by examining the left–right distribution of reported stereotactic coordinates.

## 2 Materials and Methods

### 2.1 Preregistration

This systematic review was preregistered at the international prospective register of systematic reviews (PROSPERO registration number: CRD420251172569).

### 2.2 Literature Searches

This systematic review was conducted in accordance with the Preferred Reporting Items for Systematic Reviews and Meta‐Analyses (PRISMA) guidelines. A systematic search was conducted in the electronic databases of PubMed and Web of Science Core Collection to identify relevant studies published up to August 28, 2025, using the terms “Parkinson disease”, “freezing of gait”, as well as the imaging keywords of “MRI”, “PET”, and “SPECT” (the full search strategies are provided in Table S1). We also used the AI‐assisted systematic review tool “Elicit” (Whitfield and Hofmann 2023) to retrieve publications using the research question “Which brain regions are associated with FOG in PD?”. The first step was to remove duplicates using Elicit. Next, titles, abstracts, and full texts were independently screened based on a priori defined eligibility criteria by a human reviewer (H.D.) and Elicit (default score threshold 2.5). Any disagreements were resolved by a third human reviewer (S.T.).

### 2.3 Eligibility Criteria for the Screening Process

The inclusion criteria for screening were English‐language, peer‐reviewed studies that included PD freezers and used MRI, PET, or SPECT neuroimaging. For the ALE meta‐analysis, studies were eligible if they reported peak coordinates in a standard stereotactic space (Talairach or Montreal Neurological Institute [MNI]), where the *x*‐coordinate indexed the left–right axis and the initial voxelwise search space was whole‐brain. This included subsequent group comparisons conducted within data‐driven masks derived from whole‐brain analyses. Eligible contrasts compared freezers with nonfreezers or healthy older adults. Coordinates were classified according to the direction of the reported between‐group difference and entered separately into the freezers > nonfreezers and nonfreezers > freezers ALE analyses. When multiple time points were available (e.g., longitudinal or intervention studies), only baseline outcomes were extracted. We excluded studies that did not report stereotactic coordinates, studies comparing PD to healthy older adults without specifying FOG, or studies solely reporting prespecified regions of interest (ROIs) or those that were limited to unilateral coverage. In cases where a between‐group comparison was not performed, studies reporting correlations between neuroimaging measures and FOG severity within PD freezers were retained for the descriptive summary but were not included in the ALE analyses. Based on simulation‐based recommendations for cluster‐level family‐wise error (FWE) inference, direction‐specific ALE analyses including at least 17 independent experiments were considered to meet the recommended minimum for limiting excessive influence from individual experiments, although approximately 20 experiments are desirable for adequate statistical power (Eickhoff et al. 2016). Therefore, when fewer than 17 independent experiments were available for a direction‐specific comparison, coordinates from the corresponding direct between‐group contrasts were pooled irrespective of direction in an exploratory direction‐independent ALE analysis. If significant convergence was identified in this exploratory analysis, additional direction‐specific ALE analyses were performed to characterize the direction of the observed effect. The primary ALE analyses comprised the direction‐specific freezers–nonfreezers comparisons (freezers > nonfreezers, and nonfreezers > freezers), whereas comparisons involving healthy controls were considered exploratory secondary analyses.

Based on these criteria, review articles, nonhuman studies, and non‐English publications were excluded at the title screening stage. At the abstract screening stage, nonneuroimaging studies (e.g., only behavioral/clinical data), conference abstracts, and reviews of the literature with or without meta‐analysis, nonhuman studies, non‐English, and non–peer‐reviewed articles were excluded. Studies that did not include people with PD or that explicitly did not include PD freezers, were also excluded. At the full‐text screening stage, studies were excluded for insufficient neuroimaging details, no FOG assessment, no PD freezer specifications after full‐text confirmation, no reported hemispheric right/left lateralization or stereotactic labels of brain regions/networks, or a focus on symptoms other than FOG, such as tremor, bradykinesia, and freezing of the upper limbs.

### 2.4 Data Extraction

Data were independently extracted from included studies by Elicit and a human rater (H.D.), with any discrepancies resolved by a third human reviewer (S.T.). For the descriptive summary, extracted data included study characteristics (authors, year), imaging techniques and modalities, participant demographics (sample size, age, sex), clinical characteristics (FOG severity, medication status during scan), and key brain regions in freezers. For the purpose of the ALE meta‐analysis, we extracted stereotactic coordinates (*x*, *y*, *z*) representing differences between freezers and reference groups (nonfreezers or healthy older adults). If studies reported results both with and without adjustment for confounding variables (e.g., disease duration), the adjusted coordinates were extracted. Exact duplicate foci for each experiment were removed before ALE analysis to avoid redundant weighting of nonindependent coordinates. Potentially overlapping participant samples across publications were assessed, and publications or contrasts clearly derived from the same cohort were combined and treated as a single experiment.

### 2.5 ALE Meta‐Analysis

We conducted a standard coordinate‐based meta‐analysis using the GingerALE software (version 3.0.2; www.brainmap.org/ale) to determine the above‐chance convergence of neuroimaging differences across three group comparisons: freezers versus nonfreezers, freezers versus healthy older adults, and nonfreezers versus healthy older adults. Although the primary focus was the comparison between PD freezers and nonfreezers, nonfreezers versus healthy older adults contrasts were included as exploratory secondary analyses when they were reported within the same eligible studies, to help the interpretation of the comparisons between PD freezers and nonfreezers by providing a reference for PD‐related neuroimaging differences independent of FOG. The ALE algorithm modelled coordinates as 3D Gaussian probability distributions to account for spatial uncertainty, replacing point foci with probability distributions to compute ALE scores. To reduce bias, input sample sizes were set to the lower value between the two compared groups in each contrast. Thresholding was applied following standard recommendations, utilizing a cluster‐forming threshold of uncorrected *p* < 0.001 with 5000 permutations, a cluster‐extent threshold of FWE‐corrected *p* < 0.05, and a minimum cluster extent of 200 mm^3^. This cluster‐forming and cluster‐level FWE thresholding approach is consistent with that used in previous ALE meta‐analyses (Mauchand and Zhang 2023; Heard and Lee 2020; Stefaniak et al. 2021). Studies that reported Talairach coordinates were converted to MNI space using GingerALE's conversion tool.

### 2.6 Hemispheric Lateralization

Hemispheric lateralization was evaluated based on the extracted MNI *x*‐coordinates (left: *x* < 0; right: *x* > 0; midline: *x* = 0) in the two direction‐specific freezer–nonfreezer datasets, with an alpha of 0.05. Hemispheric lateralization was primarily evaluated using a mixed‐effects logistic regression, with hemisphere (right vs. left) as the binary outcome and study/cohort unit included as a random intercept to account for within‐unit clustering of multiple foci. Midline coordinates (*x* = 0) were excluded from the binary analysis and reported descriptively. As a study‐level sensitivity analysis, a laterality index was calculated for each study/cohort unit as (right − left)/(right + left), and its deviation from zero was assessed using a two‐sided Wilcoxon signed‐rank test.

### 2.7 Risk of Bias Assessment

We developed a neuroimaging‐specific quality assessment checklist, informed by the Joanna Briggs Institute checklist (Barker et al. 2026), to evaluate reporting quality and key methodological safeguards in the included studies (Table S2). Ratings were performed by one human reviewer (H.D.) and Elicit, and any disagreements were resolved by a third human reviewer (S.T.).

## 3 Results

### 3.1 Systematic Review

Figure 1 illustrates the flow of study selection and data extraction. The screening process ultimately yielded 108 articles, of which 75 were retained for a systematic summary (details in Table S3). For the direction‐specific ALE meta‐analyses, only experiments reporting whole‐brain direct comparisons were included. The freezers > nonfreezers contrast included 18 independent experiments with 75 foci, while the nonfreezers > freezers contrast included 26 independent experiments with 118 foci. Some studies contributed eligible contrasts in both directions; therefore, these experiment counts are contrast‐specific and should not be summed to represent the number of unique studies.

**FIGURE 1:**
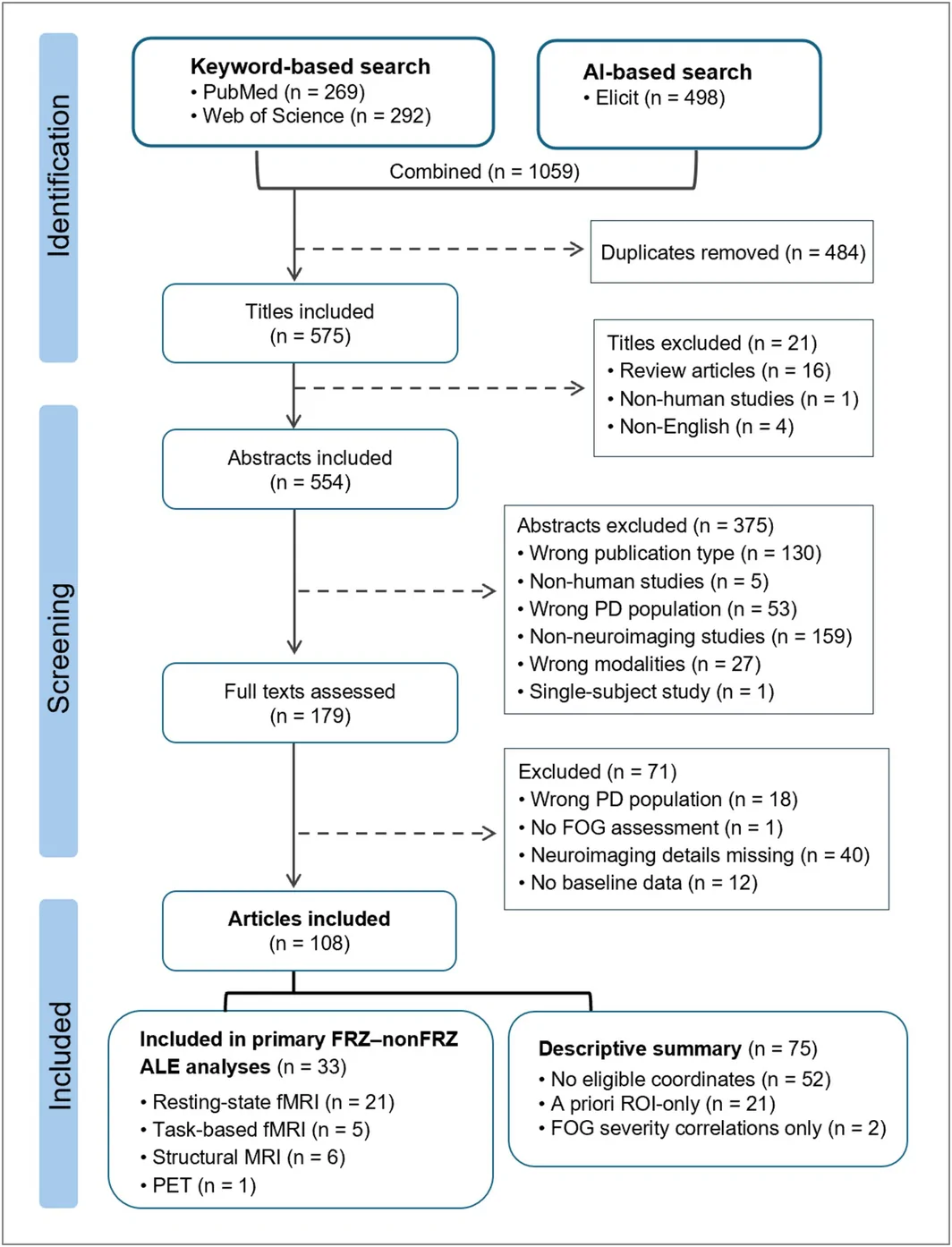
Systematic overview of selected articles. *Note:* Studies included in the ALE analyses are detailed in Table 1; studies included in the descriptive summary are detailed in Table S3. Multimodal studies were classified according to the primary modality contributing coordinates to the ALE analysis; each article was counted once. Counts in this flow diagram represent articles rather than independent experiments. Abbreviations: ALE, activation likelihood estimation; FOG, freezing of gait; PD, Parkinson's disease.

One overlapping participant cohort was identified in two publications by Lench et al. (2021) and Lench et al. (2025); these two publications were therefore treated as a single experiment. Table 1 summarizes the characteristics of studies contributing to either of the two direction‐specific ALE datasets.

**TABLE 1:** Characteristics of neuroimaging studies included in the activation likelihood estimation meta‐analysis.

Author year	Imaging technique/task^a^	Analysis type	Sample	Age	Sex, M/F	HY	UP3	FOGQ^b^	Med	LED	Key brain regions in FR compared with noFR/HA and correlations with FOG outcomes in FR^a^, hemispheric lateralization (Rt = right; Lt = left; center)
**Li Y 2020** (**Li et al.** 2020)	rs‐fMRI	ICA	25 FR 30 noFR 26 HA	66.5 60.0 60.2	15/10 17/13 11/15	2.6 2.0	29.2 25.1	10.7 1.5	On	nr	*FR* > *noFR*: FC within auditory (left temporal pole), DAN (right IFG opercular), DMN, left fronto‐parietal networks. *FR<noFR*: FC within auditory, DMN, DAN, visual networks. *Correlations*: FC in left temporal pole and right angular gyrus were positively correlated with FOGQ. Rt = 10; Lt = 5
**Li X 2025** **(Li et al.** 2025 **)**	rs‐fMRI	ALFF, fALFF, PerAF, wavelet‐ALFF	39 FR 40 noFR 44 HA	71.0 68.6 69.4	19/20 23/17 21/23	3^†^ 2.3	40 28.4/m	18.2 0.8	Off	686 376	*FR* > *noFR*: Bilateral postcentral, right IFG opercular, left precentral, left lingual, left MTG, right cerebellar VI, right crus I, vermis VIII. *FR<noFR*: Right medial orbital SFG, rectus, left cerebellar IV/V. *Correlations*: No correlation was found between FOG‐related regions and gait parameters. Rt = 16; Lt = 5
Piramide 2020 (Piramide et al. 2020)	task‐fMRI/foot movement	Whole‐brain activation	17 FR 10 noFR 18 HA	69.6 65.1 65.9	12/5 8/2 8/10	2.3 2.3	24.9 21.7	12.2	Off	nr	*FR<noFR*: Right putamen during the task. *Correlations*: FOG severity was related to reduced fronto‐parietal regions (right IFG opercular, MFG, left supramarginal); increased parieto‐occipital regions (right precuneus, superior occipital, bilateral angular gyrus). Rt = 1; Lt = 0
Yu 2021 (Yu et al. 2021)	rs‐fMRI	ICA	20 FR 21 noFR 18 HA	64.5 60.2 61.3	9/11 8/13 8/10	2.4 2.0	21.0 18.9	14.1 4.0	Off	488 470	*FR* > *noFR*: FC within DMN (bilateral MFG, left DLPFC). *FR<noFR*: FC within sensory‐motor network (right precentral gyrus, SMA), medial visual network (right precuneus, calcarine), lateral visual network (bilateral lingual gyrus, cuneus). Rt = 4; Lt = 2
Taniguchi 2024 (Taniguchi et al. 2024)	rs‐fMRI	Seed‐voxel FC, dynamic causal modelling, VBM	41 FR 41 noFR	69.1 67.2	20/21 22/19	nr	37.9 26.2/m	15.5 7.8	On	492 282	*FR* > *noFR*: FC between bilateral MLR and DLPFC. Excitatory effective connectivity from left DLPFC to bilateral MLR, self‐connection within left DLPFC. *Correlations*: These were inversely related to FOG severity and were independent of GM volumes. Rt = 2; Lt = 2
Lench 2020 (Lench et al. 2020)	rs‐fMRI	Seed‐voxel FC	27 FR 27 noFR	67.2 67.9	20/7 22/5	nr	17.8 18.2	20.3 /N	On	1200 651	*FR* > *noFR*: FC between left MLR/PPN and bilateral midcingulate cortex, left supramarginal, and left temporal pole. *Correlations*: These FCs positively correlated with off‐state objective gait measures. Rt = 1; Lt = 2
Guo 2020 (Guo et al. 2020)	rs‐fMRI	Graph theory, seed‐voxel FC	22 FR 28 noFR 33 HA	61.3 62.8 63.1	13/9 16/12 13/20	1.96 2.09	28 28.1	11.1 1.2	Off	479 431	*FR* > *noFR*: Degree centrality in right middle frontal gyrus (midFG), FC of right middle frontal gyrus —precuneus, —cerebellum. *FR<noFR*: FC between right midFG and IFG/SFG. *Correlations*: FOGQ was negatively associated with degree centrality in right midFG, and FC of right midFG—cerebellum. Rt = 5; Lt = 0
Sun 2025 (Sun et al. 2025)	rs‐fMRI (d‐MRI)	Seed‐voxel FC, (microstructure)	110 FR 79 noFR 52 HA	68^†^ 67 64	74/36 53/26 33/19	2^†^ 2	32^†^ 32/ m	19^†^ /N	On and off	681 663	*FR<noFR*: FC of locus coeruleus —right occipital regions (lingual, calcarine). (In microstructure, FR showed distinct signs of tissue degeneration in locus coeruleus. *Correlations*: Free water and axial diffusivity were positively related to NFOGQ). Rt = 2; Lt = 0
Wang S 2021 (Wang et al. 2021)	rs‐fMRI	Seed‐voxel FC, dynamic FC	25 FR 25 noFR 25 HA	65.3 65.2 63.9	13/12 13/12 11/14	2.6 2.5	33.3 33.0	20.5 /N	Off	466 378	*FR* > *noFR and HA*: Dynamic FC of left intralaminar nuclei—right IPL, left medial geniculate nuclei—left PCG. *FR and HA<noFR*: Dynamic FC of right ventral lateral nuclei— left PCL. *Correlations*: Dynamic FC of left intralaminar nuclei—right IPL was positively correlated with NFOGQ. Rt = 1; Lt = 2
Zhou 2018 (Zhou et al. 2018)	rs‐fMRI	Regional homogeneity	14 FR 20 noFR 18 HA	62.1 62.7 63.7	9/5 11/9 10/8	3.2 2.3	35.5 35.5	nr	Off	nr	*FR<noFR*: Regional homogeneity values within left SMA and left superior frontal region; *FR<HA and noFR<HA*: Regional homogeneity values within left SMA and left superior frontal region; *FR* > *HA; noFR* > *HA*: Regional homogeneity values within right putamen. Rt = 0; Lt = 1; center = 1
Wang M 2016 (Wang et al. 2016)	rs‐fMRI (d‐MRI)	Seed‐voxel FC, (microstructure)	14 FR 16 noFR 16 HA	72.4 68.9 68.6	9/5 10/6 8/8	nr	24.6 20.9	16.1 1.6	Off	401 344	*FR<noFR and HA*: FC between right PPN and right MTG, between right ITG and bilateral cerebellum. *FR* > *noFR*: FC of left PPN—right MTG. *In microstructure FR* vs. *HA*: FR had both increased mean diffusivity and decreased FA in multiple regions including left cingulum, corpus callosum. Rt = 5; Lt = 2
Kou 2022 (Kou et al. 2022)	rs‐fMRI (d‐MRI)	Seed‐voxel FC (microstructure)	24 FR 22 noFR 27 HA	62.6 63.8 63.8	11/13 11/11 14/13	3^†^ 3	39.7 39.0/m	nr	Off	900^†^ 880	*FR<noFR*: FC between right GPi and left middle occipital gyrus, and right calcarine. (*FR* > *noFR and HA*: In microstructure, mean diffusivity and radial diffusivity values in right GPi.) Rt = 1; Lt = 1
Tessitore 2012 (Tessitore, Amboni, Esposito, et al. 2012)	rs‐fMRI	ICA	16 FR 13 noFR 15 HA	66.9 66.3 65.5	12/4 9/4 11/4	1.8 1.8	17.5 13.0	11.7 2.3	On	540 552	*FR<noFR*: FC within both executive‐attention (in right MFG and in angular gyrus) and visual networks (in right occipito‐temporal gyrus). *Correlations*: Individual ROI‐averaged ICA scores were negatively correlated with FOGQ. Rt = 3; Lt = 0
Tessitore 2012 (Tessitore, Amboni, Cirillo, et al. 2012)	s‐MRI	VBM	12 FR 12 noFR 12 HA	66.8 66.4 65.5	8/4 8/4 7/5	1.8 1.7	18.0 12.8	12.2 3.4	n/a	603 580	*FR<noFR*: GM volume in left cuneus, precuneus, lingual gyrus, posterior cingulate cortex. *Correlations*: FOGQ was negatively correlated with GM volume in left cuneus. Rt = 1; Lt = 3
Mi 2017 (Mi et al. 2017)	rs‐fMRI	ALFF	31 FR 31 noFR 32 HA	60.9 58.0 58.3	14/17 20/11 17/15	2.6 1.9	41.5 30.8/m	II 15.1 III 5.8/N	Off	705 397	*FR* > *noFR*: Right ACC and left IPL. *FR<noFR*: right SFG, bilateral cerebellum, left thalamus. *Correlations*: FOG severity (NFOGQ, gait) positively correlated with ALFF in right SFG, ACC, bilateral pallidum; negatively in thalamus, putamen, cerebellum, sensorimotor regions. Rt = 3; Lt = 3
Lenka 2016 (Lenka et al. 2016)	rs‐fMRI	Seed‐voxel FC	15 FR 13 noFR 30 HA	54.5 50.3 52.5	10/5 9/4 20/10	1.8 1.6	36.8 32.0	12.7 2.6	Off	637 578	*FR<noFR*: Interhemispheric FC primarily between left parietal opercular cortex and multiple regions including contralateral somatosensory, auditory, and opercular areas. Rt = 0; Lt = 4
Hu 2020 (Hu et al. 2020)	rs‐fMRI	ALFF	13 FR 15 noFR 17 HA	60.8 60.9 61.0	9/4 9/6 11/6	2.9 2.1	36.5 34.6	nr	Off	nr	*FR* > *noFR and HA*: Left ITG in slow‐4 band, left ITG/MTG in the classical band. *FR<noFR*: Right MFG specifically in slow‐4 band. *FR and noFR<HA*: Bilateral putamen in slow‐5 band. Rt = 1; Lt = 3
Bharti 2020 (Bharti et al. 2020)	rs‐fMRI	ICA	15 FR 16 noFR 16 HA	71.0 66.3 66.7	12/3 13/3 10/6	2.3 2	36.2 29	nr	Off	nr	*FR* > *noFR*: Within‐network FC in orbitofrontal, frontal, sensorimotor, right fronto‐parietal, BG networks regions. *FR<HA*: FC of right fronto‐parietal—executive‐control networks. Correlations: The FC of right fronto‐parietal—executive‐control network was negatively associated with FOGQ. Rt = 5; Lt = 2
Wang S 2022 (Wang et al. 2022)	rs‐fMRI	Whole‐brain cerebral blood flow	30 FR 30 noFR 30 HA	64.1 60.7 62.9	15/15 13/17 16/14	2.6 2.2	31.2 28.9	21.3 /N	Off	420 341	*FR* > *noFR*: Bilateral thalamus and left caudate. *FR<noFR*: Left IPL. *Correlations*: Cerebral blood flow in left caudate was positively associated with NFOGQ. Rt = 1; Lt = 3
Lench 2021 (Lench et al. 2021)	rs‐fMRI	Seed‐voxel FC	38 FR 17 noFR	67.1 68.6	27/11 14/3	2.1 2.0	18.2 18.6	20.4 /N	On	1182 510	Exp.1 (baseline). *FR* > *noFR*: FC between left SMA and vermis of cerebellum. *FR<noFR*: FC between SMA and orbitofrontal cortex. Combined foci with Lench 2025: Rt = 5; Lt = 9.
Lench 2025 (Lench et al. 2025)	rs‐fMRI	Seed‐voxel FC	38 FR 17 noFR	67.1 68.6	27/11 14/3	2.1 2.0	18.2 18.6	20.4 /N	On	1182 510	*FR* > *noFR*: FC of vermis—SPL, SMA, postcentral gyrus, posterior cingulate cortex. *FR<noFR*: FC of vermis—occipital cortex. *Correlations*: Reduced gait automaticity was related to increased FC of vermis—sensorimotor cortex, —SPL. See combined foci above.
Steidel 2021 (Steidel et al. 2021)	PET and rs‐fMRI	Voxel‐wise and seed‐voxel FC, ROI‐wise FC	27 FR 32 noFR	66.5 64.4	17/10 23/9	2.4 2.2	27.2 22.4	10.2 2.0	Off	518 458	*FR<noFR*: Dopamine metabolism in left caudate nucleus; Molecular connectivity of bilateral ventral tegmental area—ACC, right parahippocampus, amygdala, ventromedial PFC. *FR* > *noFR*: FC of left caudate nucleus— right visual cortex. FC of bilateral ventral tegmental area—DMN regions (precuneus, angular gyrus, SFG, superior lateral occipital cortex). Rt = 4; Lt = 2
Droby 2021 (Droby et al. 2021)	rs‐fMRI and s‐MRI	VBM with Seed‐voxel FC	16 FR 15 noFR 20 HA	72 68.2 64	11/5 10/5 9/11	2.5 1.8	27.9 18.9/m	16.44	On	nr	*FR<noFR*: FC of midbrain—middle cingulate cortex. *FR<HA*: FC of midbrain—parieto‐temporal regions, specifically right postcentral gyrus, supramarginal, and superior temporal gyrus. Rt = 3; Lt = 2
Snijders 2011 (Snijders et al. 2011)	task‐fMRI/imagery and s‐MRI	Whole‐brain activation, VBM	12 FR 12 noFR 21 HA	58.7 62.6 57.0	8/4 7/5 12/9	nr	34.6 28.6	12.4^†^ /N	Off	nr	*FR* > *noFR* (motor > visual imagery): Activation in MLR (x = 0). *FR<noFR* (motor > visual imagery): Activation in left SMA and right SPL. Structurally, FR showed GM atrophy in MLR (x = 2). center = 1
Gilat 2015 (Gilat et al. 2015)	task‐fMRI/VR	Whole‐brain, ROI‐to‐ROI	17 FR 10 noFR	67.4 64.8	nr	2.5^†^ 2	37.2 30.1	3.1^†^ /item3	Off	776 785	*FR<noFR*: Left SMA, premotor area, SPL. *FR* > *noFR*: Bilateral IFG during turning, FC within subcortical locomotor network (STN, GPi, MLR, CLR) during turning. Rt = 1; Lt = 3
Shine 2013 (Shine et al. 2013)	task‐fMRI/VR	Whole‐brain activation	14 FR 15 noFR	63.2 63.4	nr	2.2 1.9	31.9 29.1	10.5 1.7	Off	nr	*FR<noFR*: Bilateral anterior insula, ventral striatum, pre‐SMA, and left STN during the processing of increased cognitive load (indirect vs. direct cues) while performing the VR task. Rt = 3; Lt = 4
Xie 2025 (Xie et al. 2025)	task‐fMRI/cognitive task	Whole‐brain activation, Group ICA	22 FR 20 noFR 22 HA	62.0 61.7 63.1	14/8 14/6 9/13	2.9 2.8	20.2 16.3	17.3 1.2	On Exp.2	nr	*FR* > *noFR and HA*: Dorsal ACC, right MFG, bilateral MTG, posterior cingulate cortex during conflict resolution (incongruent > congruent). *Correlations*: FOGQ was positively correlated with dorsal ACC, left MTG, posterior cingulate cortex. Rt = 3; Lt = 1; center = 1
Camastra 2025 (Camastra et al. 2025)	s‐MRI	VBM	86 FR 394 noFR	64.6 62.7	59/27 223/171	1.9 1.6	25.8 21.3	2.7/m item	n/a	nr	*FR<noFR*: GM volume in bilateral cerebellar lobes, bilateral hippocampus, right putamen, left insula, left inferior temporal gyrus, left lateral orbitofrontal gyrus. Rt = 3; Lt = 6
Rubino 2014 (Rubino et al. 2014)	s‐MRI	VBM	13 FR 13 noFR	68.1 68.7	10/3 9/4	2.4 2.3	17.9 17.2	13.1 3.6	n/a	755 507	*FR<noFR*: GM volume in left PPC. Rt = 0; Lt = 1
Jha 2015 (Jha et al. 2015)	s‐MRI	VBM	17 FR 21 noFR	56.9 47.4	11/6 11/10	1.96 1.78	24.3 12.6	13.6	n/a	563 540	*FR<noFR*: GM in right cerebellum, left MTG, right medial frontal, precentral gyrus. *Correlations*: FOGQ was negatively correlated with GM in right medial frontal, lingual, left postcentral gyrus, IPL. Rt = 4; Lt = 2
Vaštík 2017 (Vastik et al. 2017)	s‐MRI	Vertex‐wise, ROI based GM	11 FR 10 noFR	71.3 68.7	9/2 4/6	3.4^c^ 2.9	29.8 20.6	15.9	n/a	nr	*FR<noFR*: Cortical thickness in bilateral cingulate cortex (midanterior and posterior), left SMA, right frontal operculum, and left temporal pole. Rt = 1; Lt = 3
Kostic 2012 (Kostić et al. 2012)	s‐MRI	Whole‐brain VBM	17 FR 20 noFR 34 HA	64 63 64	20/14 12/8 27/7	2.7 2.4	39 29	16 5	n/a	804 716	*FR<noFR*: GM volume in left IFG, left precentral gyrus, and left inferior parietal gyrus. *Correlations*: FOGQ was negatively correlated with GM volume in left SFG/MFG/IFG, right SFG, middle/posterior cingulate gyri. Rt = 0; Lt = 3
Tard 2015 (Tard et al. 2015)	PET	Whole‐brain voxel‐wise	11 FR 11 noFR	61.4 62.2	7/4 8/3	nr	34.6 25.4	12.9 0.2	Off	1129 807	*FR<noFR*: Associative premotor cortex, temporopolar/orbitofrontal area, left DLPFC. *FR* > *noFR*: GP, left PPC, PCL. *Correlations*: FOG time percentage was positively correlated with activity in cerebellum, PCL, and right frontal eye field, and negatively correlated with activity in orbitofrontal area, premotor cortex, left SMA, and temporal lobe. Rt = 14; Lt = 11

*Note:* The top two studies contributing to the main ALE cluster are highlighted in bold. Data are shown as means unless otherwise indicated († median). Rt/Lt/center counts descriptively summarize the hemispheric distribution of foci from freezer–nonfreezer comparisons, irrespective of the direction of the reported effect. Overlapping cohorts were counted once.

Abbreviations: ACC, anterior cingulate cortex; ALFF, amplitude of low‐frequency fluctuation; BG, basal ganglia; CLR, cerebellar locomotor region; DAN, dorsal attention network; DMN, default mode network; DLPFC, dorsolateral prefrontal cortex; d‐MRI, diffusion MRI; FA, fractional anisotropy; FC, functional connectivity; FOGQ, Freezing of Gait Questionnaire; FR, freezers; GM, grey matter; GP, globus pallidus; GPi, internal globus pallidus; HA, healthy older adults; HY, Hoehn and Yahr stage; ICA, independent component analysis; IFG, inferior frontal gyrus; IPL, inferior parietal lobule; ITG, inferior temporal gyrus; LED, levodopa equivalent daily dose; Med, medication status during scan; MFG, middle frontal gyrus; MLR, mesencephalic locomotor region; MTG, middle temporal gyrus; noFR, nonfreezers; n/a, not applicable; nr, not reported; PCA, principal component analysis; PCG, postcentral gyrus; PCL, paracentral lobule; PFC, prefrontal cortex; PPC, posterior parietal cortex; PPN, pedunculopontine nucleus; ROI, region of interest; SFG, superior frontal gyrus; SMA, supplementary motor area; SPL, superior parietal lobule; s‐MRI, structural MRI; STN, subthalamic nucleus; UP3, Unified Parkinson's Disease Rating Scale Part III; VBM, voxel‐based morphometry; VR, virtual reality gait task.

^a^ If applicable.

^b^ Scores represent the Freezing of Gait Questionnaire (FOGQ); ‘N' indicates the New Freezing of Gait Questionnaire, and ‘m’ indicates the Movement Disorder Society‐Sponsored Revision of the Unified Parkinson's Disease Rating Scale Part III.

^c^ Values were calculated from category distributions.

### 3.2 Quality Assessment

Table S2 summarizes the quality assessment of the 33 studies included in the ALE meta‐analysis. Freezer/nonfreezer classification and neuroimaging methods were clearly described in all studies, and FOG severity was appropriately described in 32 studies (97.0%). PD diagnostic criteria and disease severity were reported in 29 studies (87.9%), key confounders were addressed in 30 studies (90.9%), and preprocessing procedures were described in sufficient detail in 30 studies (90.9%). In contrast, head‐motion reporting was less consistent, with 21 studies (63.6%) quantitatively reporting head motion and 21 studies (63.6%) describing motion‐correcting strategies. Medication status during scanning was clearly described in 30 studies (90.9%). Among the 22 studies that examined correlations between neuroimaging findings and FOG severity in PD freezers, 16 used FOG severity measures independent of the freezer/nonfreezer classification metric, whereas 6 did not. The remaining 11 of 33 studies did not examine such clinical correlations.

### 3.3 Hemispheric Lateralization

Table 2 summarizes the hemispheric distribution of foci. The primary mixed‐effects logistic regression showed no evidence of hemispheric lateralization in either direction‐specific dataset. The study‐level sensitivity analysis yielded consistent results, with the laterality index not differing significantly from zero in either contrast.

**TABLE 2:** Hemispheric distribution of extracted coordinates for the comparison between freezers and nonfreezers.

				Montreal Neurological Institute *x*‐coordinates			Hemispheric lateralization		
	Contrast	*N*	Foci	Right (*x* > 0)	Left (*x* < 0)	Center (*x* = 0)	Odds ratio (95% CI)	Median laterality index (IQR)	*p*
Primary mixed‐effects analysis	Freezers > nonfreezers	18	75	42	31	2	1.33 (0.80 to 2.21)	—	0.278
	Nonfreezers > freezers	26	118	61	56	1	1.08 (0.67 to 1.72)	—	0.759
Study‐level sensitivity analysis	Freezers > nonfreezers	17^a^	—	7	5	5	—	0.00 (−0.33 to 0.47)	0.344
	Nonfreezers > freezers	26	—	11	12	3	—	0.00 (−0.88 to 0.62)	0.793

*Note:* In the primary analysis, odds ratios for right‐sided versus left‐sided foci and corresponding *p*‐values were derived from mixed‐effects logistic regression models, with hemisphere (right vs. left) as the binary outcome and study/cohort unit included as a random intercept to account for within‐unit clustering of multiple foci. Midline coordinates (*x* = 0) were excluded from the binary models and are reported descriptively. In the study‐level sensitivity analysis, the laterality index was calculated for each study/cohort unit as (right − left)/(right + left), with positive and negative values indicating right‐ and left‐hemispheric predominance, respectively. For the primary mixed‐effects analysis, *N* denotes the number of independent ALE experiments, with eligible contrasts derived from the same participant sample combined into a single experiment. For the study‐level sensitivity analysis, *N* denotes the corresponding study/cohort units after exclusion of units for which the laterality index could not be calculated. For the study‐level sensitivity analysis rows, the right, left, and center columns indicate the numbers of right‐predominant, left‐predominant, and equal study/cohort units, respectively. Deviation of the laterality index from zero was assessed using a two‐sided Wilcoxon signed‐rank test.

^a^ One study‐level unit containing only a midline focus (*x* = 0) was excluded from the study‐level laterality index analysis because the laterality index could not be calculated.

### 3.4 Peak Cluster Coordinates in the ALE Meta‐Analysis

Figure 2 shows the significant clusters identified in the primary and exploratory ALE analyses. Primary ALE analyses were conducted for the freezers > nonfreezers (18 independent experiments) and nonfreezers > freezers (26 independent experiments) contrasts. Because fewer than 17 independent experiments were available for comparisons involving healthy controls, these secondary comparisons were evaluated using exploratory ALE analyses.

**FIGURE 2:**
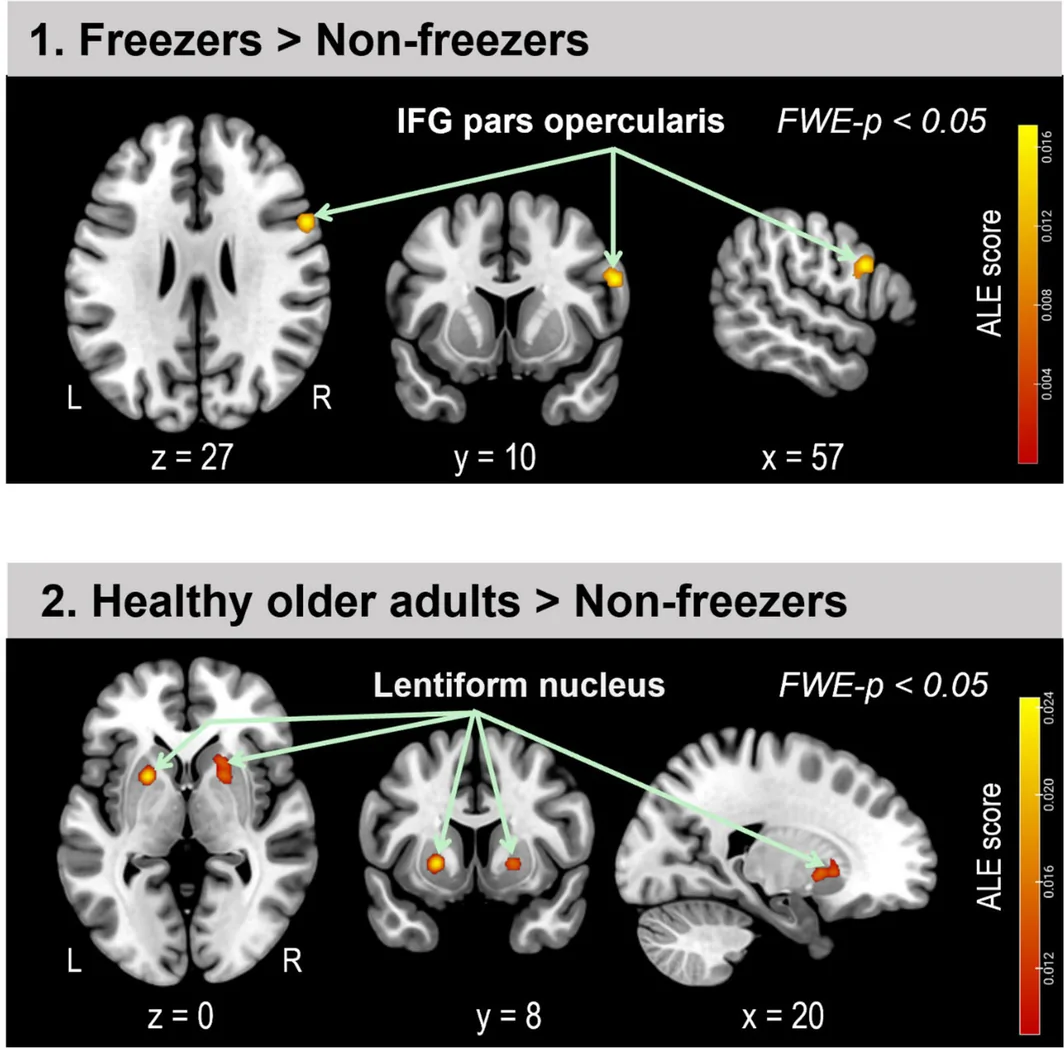
Significant ALE meta‐analysis results for the freezers > nonfreezers and healthy older adults > nonfreezers contrasts. (1) In the freezers > nonfreezers contrast, the main ALE analysis at cluster‐level FWE‐corrected *p* < 0.05 revealed a significant cluster of convergence in the right inferior frontal gyrus. The peak MNI coordinates were at *x* = 57, *y* = 10, *z* = 27. (2) In the healthy older adults > nonfreezers contrast, the additional ALE analysis at cluster‐level FWE‐corrected *p* < 0.05 revealed two significant clusters in the bilateral lentiform nuclei. The peak MNI coordinates were *x* = 20, *y* = 8, *z* = 0 on the right; *x* = −22, *y* = 8, *z* = 0 on the left. *Note:* Images were displayed in neurological convention using MRIcroGL (https://www.nitrc.org/projects/mricrogl/), with the left hemisphere shown on the left and the right hemisphere on the right.

#### 3.4.1 Primary ALE Analyses Between Freezers Versus Nonfreezers

##### 3.4.1.1 Freezers > Nonfreezers

The freezers > nonreezers analysis included 75 extracted foci from 18 independent experiments comprising 846 participants and was performed using cluster‐level FWE correction at *p* < 0.05. The cluster‐level FWE procedure yielded a cluster‐extent threshold of 584 mm^3^, and one cluster survived this threshold in the right inferior frontal gyrus, pars opercularis (IFG; predominantly within Brodmann area 9 by functional label). The peak MNI coordinates were *x* = 57, *y* = 10, *z* = 27 (cluster size = 736 mm^3^; ALE value = 0.0171, *Z* = 4.62; Figure 2, panel 1). Two of the 18 independent experiments contributed foci to this cluster (Li et al. 2020; Li et al. 2025). Both were resting‐state fMRI studies and reported greater right IFG involvement in freezers than in nonfreezers. The medication states differed between the two studies: Li et al. (2020) assessed patients in the ON‐medication state (Li et al. 2020), whereas Li et al. (2025) assessed patients in the OFF‐medication state.

##### 3.4.1.2 Nonfreezers > Freezers

The nonfreezers > freezers analysis included 118 extracted foci from 26 independent experiments comprising 1593 participants and was performed using cluster‐level FWE correction at *p* < 0.05; no significant clusters were identified.

#### 3.4.2 Exploratory ALE Analyses Between Nonfreezers and Healthy Older Adults

This exploratory analysis included 10 studies (504 subjects, 70 foci) and revealed two significant clusters in the bilateral lentiform nuclei, mainly putamen (cluster‐level FWE‐corrected *p* < 0.05, 5000 permutations). The peak MNI coordinates were located in the right lentiform nucleus (*x* = 20, *y* = 8, *z* = 0; ALE value = 0.0158, *Z* = 4.39) and the left lentiform nucleus (*x* = −22, *y* = 8, *z* = 0; ALE value = 0.0245, *Z* = 5.77). Four studies primarily contributed to these clusters (Li et al. 2025; Mi et al. 2017; Hu et al. 2020; Wang et al. 2022). All were resting‐state fMRI studies conducted in the OFF‐medication state and consistently reported reduced involvement of these regions in PD nonfreezers compared with healthy older adults.

In the direction‐specific follow‐up analysis, the bilateral lentiform nucleus clusters identified in the direction‐independent analysis were also observed in the healthy older adults > nonfreezers contrast, indicating greater involvement in healthy older adults than in nonfreezers. This analysis included 9 independent experiments (448 participants, 43 foci) and revealed two significant clusters. The right cluster (1408 mm^3^) peaked at MNI coordinates *x* = 20, *y* = 8, *z* = 0 (ALE = 0.0158, *Z* = 4.61) and predominantly involved the lentiform nucleus, particularly the putamen, with additional caudate involvement. The left cluster (816 mm^3^) peaked at *x* = −22, *y* = 8, *z* = 0 (ALE = 0.0243, *Z* = 6.02) and was localized to the left putamen. These findings are shown in Figure 2, panel 2. Four studies primarily contributed to these clusters (Li et al. 2025; Mi et al. 2017; Hu et al. 2020; Wang et al. 2022). All were resting‐state fMRI studies conducted in the OFF‐medication state and consistently reported reduced involvement of these regions in PD nonfreezers compared with healthy older adults.

#### 3.4.3 Exploratory ALE Analyses Between Freezers and Healthy Older Adults

This exploratory analysis included 11 studies (495 subjects, 110 foci) and was performed using cluster‐level FWE correction at *p* < 0.05. No significant clusters were identified.

## 4 Discussion

This systematic review and ALE meta‐analysis synthesized coordinate‐based neuroimaging evidence to identify convergent brain regions related to FOG in PD and examine possible hemispheric lateralization underlying FOG.

In relation to our primary objective of identifying brain regions most consistently associated with FOG, the descriptive synthesis of the included studies showed that FOG‐related alterations were reported across a widely distributed set of cortical, subcortical, cerebellar, and brainstem regions. These findings involved frontal control regions, parietal and occipital visuospatial–attentional areas, basal ganglia–thalamic circuits, cerebellar regions, and brainstem locomotor‐related structures, reflecting substantial heterogeneity across study settings, imaging modalities, analytical approaches, and task conditions. This distributed pattern is consistent with the current network‐based conception of FOG (Weiss et al. 2020).

The ALE analysis further revealed the right IFG as the region most consistently associated with FOG in PD. Both contributing studies reported greater right IFG involvement in freezers than in nonfreezers at rest (Li et al. 2020; Li et al. 2025). Hypothetically, greater right prefrontal activity in freezers may reflect an increased reliance on top–down cognitive control as gait becomes less automatic (Vitorio et al. 2020; Pu et al. 2023; Vandenbossche et al. 2013); whether this recruitment is compensatory, maladaptive, or an unsuccessful compensation remains unresolved. However, this interpretation is consistent with evidence at the broader prefrontal level rather than specifically localizing it to the right IFG. Despite the localized convergence in the right IFG, this finding should not be interpreted as evidence of a focal abnormality in freezers. Rather, the right IFG might also act as a frontal control hub within a broader cortico‐subcortical network, through which cognitive control, visuospatial attentional, and locomotor processes interact. This interpretation is supported by the current network‐based view of FOG, in which freezing arises from integration failure across distributed cortical and subcortical nodes rather than from dysfunction of a single isolated region (Weiss et al. 2020).

In our additional nonfreezers versus healthy older adults comparison, bilateral lentiform nucleus alterations were observed, consistent with core basal ganglia dysfunction related to PD motor symptoms (Bhatia and Marsden 1994). These findings suggest that lentiform nucleus alterations may reflect broader PD motor‐related pathology, rather than a FOG‐specific differentiating feature. Together with the right IFG findings that were identified in the freezer–nonfreezer comparison, the absence of striatal differences emphasizes that basal ganglia circuit changes alone do not fully explain FOG, as previously suggested (Bohnen et al. 2014). Unlike the nonfreezer–healthy older adult comparison, the freezer–healthy older adult comparison revealed no significant convergence. This lack of convergence is consistent with the spatially heterogeneous pattern reported across individual studies directly comparing freezers with healthy older adults, which have implicated distributed cortical, subcortical, cerebellar, and brainstem regions rather than a common focal region (Kostić et al. 2012; Droby et al. 2021; Bharti et al. 2020). Further studies are therefore required to clarify the neural alterations distinguishing freezers from healthy older adults.

As summarized in Table 2, we found no evidence of hemispheric lateralization in the distribution of extracted foci in either direction‐specific dataset. This finding does not provide evidence for overall hemispheric lateralization, consistent with previous reports questioning a strongly unilateral account of FOG (Bardakan et al. 2022; Hall et al. 2015; Fasano et al. 2015). Nevertheless, when accounting for sample size and spatial uncertainty using the rigorous ALE meta‐analysis, a significant convergent cluster emerged in the right IFG (pars opercularis). Although the right IFG was the most consistently identified region, our broader descriptive evaluation and synthesis did not reveal clear hemispheric lateralization. Therefore, the findings of this review do not support a strongly predominant right‐sided hemispheric lateralization underlying FOG in PD.

### 4.1 Strengths and Limitations

To the best of our knowledge, this is the first ALE meta‐analysis to identify consistent brain regions differentiating freezers from nonfreezers in people with PD. We could include a reasonably large number of studies in the ALE analysis. Nevertheless, the included studies were heterogeneous in terms of imaging modality, task paradigm, medication state, FOG severity, sample size, and analytical covariates. Therefore, further research is needed to clarify the neurophysiological interpretation and modality‐specific nature of the convergent right IFG finding. In particular, medication state varied across the studies contributing to the main ALE cluster, which may have influenced both neural activity and FOG severity and thereby contributed to between‐study heterogeneity. Future studies should therefore consider medication state more systematically, including separate analyses of ON‐ and OFF‐medication conditions.

## 5 Conclusion

This systematic review and ALE meta‐analysis identified a significant convergence of regional alterations in the right IFG in freezers compared with nonfreezers. Although we found no evidence for overall hemispheric lateralization, the right IFG may represent a key frontal control hub within broader networks implicated in FOG.

## Author Contributions

**Seira Taniguchi:** methodology, writing – original draft, writing – review and editing, validation, formal analysis, funding acquisition, data curation, software, investigation, visualization, conceptualization, project administration. **Hannah Deckmyn:** writing – review and editing, data curation, methodology, investigation. **Moran Gilat:** conceptualization, methodology, supervision, writing – review and editing.

## Ethics Statement

Not applicable. This study was a systematic review and meta‐analysis of previously published data and did not involve the recruitment of human participants.

## Conflicts of Interest

The authors declare no conflicts of interest.

## Supporting information


**Table S1:** Search strategy.
**Table S2:** Quality of neuroimaging studies included in the activation likelihood estimation (ALE) meta‐analysis.
**Table S3:** Characteristics of studies included in narrative synthesis.

## Data Availability

The data supporting the findings of this study are available from the corresponding author upon reasonable request.
