# Supplementary material for: Inferior Frontal Gyrus Alterations in Freezing of Gait in Parkinson's Disease Without Evidence for Overall Hemispheric Lateralization: A Systematic Review and Activation Likelihood Estimation Meta‐Analysis
Source: Eur J Neurosci. 2026 Oct 29;64(9):e70739. doi: 10.1111/ejn.70739 (PMC13719531; doi:10.1111/ejn.70739)
Supplement: Supplementary file 1 [file EJN-64-0-s001.docx]

**Table S1. Search strategy**

| **Database** | **Search strategy** |
| --- | --- |
| PubMed | (("parkinson disease"[MeSH Terms] OR "parkinson*"[All Fields]) AND (("freezing of gait"[All Fields] OR "freezing" OR ("freez*"[All Fields] AND ("gait"[MeSH Terms] OR "gait"[All Fields] OR "walk*"[All Fields] OR "step*"[All Fields]))) AND ("magnetic resonance imaging"[MeSH Terms] OR "mri"[All Fields] OR ("positron emission tomography"[MeSH Terms] OR "positron emission tomography"[All Fields] OR "pet"[All Fields]) OR ("tomography, emission computed, single photon"[MeSH Terms] OR "single-photon emission-computed tomography"[All Fields] OR "spect"[All Fields])))) |
| Web of Science | ("Parkinson disease" OR Parkinson*) AND (("freezing of gait" OR "freezing" OR (freez* AND (gait OR walk* OR step*))) AND (("magnetic resonance imaging" OR "mri") OR ("positron emission tomography" OR "pet") OR ("single photon emission computed tomography" OR "single-photon emission computed tomography" OR "spect"))) |
| Elicit | The following research question was entered:  “Which brain regions are associated with freezing of gait in Parkinson’s disease?” |

**Table S2. Quality of neuroimaging studies included in the activation likelihood estimation (ALE) meta-analysis.**

n/a = not applicable, Med status = medication status. Q1. Were the criteria for diagnosis and disease severity in PD clearly described?; Q2. Was the method used to classify PD freezers and non-freezers clearly described?; Q3. Was the severity of FOG in freezers appropriately described?; Q4. Were neuroimaging methods described in sufficient detail?; Q5. Were key confounders measured and, if needed, adjusted statistically?; Q6. Were neuroimaging preprocessing procedures described?; Q7. Was head motion during neuroimaging acquisition measured and reported?; Q8. Were the strategies to deal with head motion stated?; Q9. Was the ON/OFF-medication status during scanning clearly described?; Q10. (If applicable) For correlations between FOG severity and neuroimaging results in freezers, were FOG severity measures different from (i.e., independent of) the metrics used to classify freezers and non-freezers? NOTE: Not applicable (n/a) was selected for studies that did not examine correlations

|  | Q1. PD criteria | | Q2. Freezer classification | | Q3. FOG severity | | Q4. Imaging methods | | Q5. Confounders | | Q6. Preprocessing | | Q7. Head motion reports | | Q8. Motion handling | | Q9. Med status | | Q10. Independent FOG measure | | |
| --- | --- | --- | --- | --- | --- | --- | --- | --- | --- | --- | --- | --- | --- | --- | --- | --- | --- | --- | --- | --- | --- |
| Author, Year | Yes | No | Yes | No | Yes | No | Yes | No | Yes | No | Yes | No | Yes | No | Yes | No | Yes | No | Yes | No | n/a |
| Li Y 2020 (1) | 1 |  | 1 |  | 1 |  | 1 |  | 1 |  | 1 |  | 1 |  | 1 |  | 1 |  | 1 |  |  |
| Li X 2025 (2) | 1 |  | 1 |  | 1 |  | 1 |  | 1 |  | 1 |  | 1 |  | 1 |  | 1 |  | 1 |  |  |
| Piramide 2020 (3) |  | 1 | 1 |  | 1 |  | 1 |  | 1 |  | 1 |  |  | 1 |  | 1 | 1 |  | 1 |  |  |
| Yu 2021 (4) | 1 |  | 1 |  | 1 |  | 1 |  | 1 |  | 1 |  | 1 |  | 1 |  | 1 |  |  |  | 1 |
| Taniguchi 2024 (5) | 1 |  | 1 |  | 1 |  | 1 |  | 1 |  | 1 |  | 1 |  | 1 |  | 1 |  | 1 |  |  |
| Lench2020 (6) | 1 |  | 1 |  | 1 |  | 1 |  |  | 1 | 1 |  | 1 |  | 1 |  | 1 |  | 1 |  |  |
| Guo 2020 (7) | 1 |  | 1 |  | 1 |  | 1 |  | 1 |  | 1 |  | 1 |  | 1 |  | 1 |  | 1 |  |  |
| Sun 2025 (8) | 1 |  | 1 |  | 1 |  | 1 |  | 1 |  | 1 |  | 1 |  | 1 |  | 1 |  | 1 |  |  |
| Wang S 2021(9) | 1 |  | 1 |  | 1 |  | 1 |  | 1 |  | 1 |  | 1 |  | 1 |  | 1 |  | 1 |  |  |
| Zhou 2018 (10) | 1 |  | 1 |  | 1 |  | 1 |  | 1 |  | 1 |  | 1 |  | 1 |  | 1 |  |  |  | 1 |
| Wang M 2016 (11) | 1 |  | 1 |  | 1 |  | 1 |  | 1 |  | 1 |  | 1 |  | 1 |  | 1 |  |  |  | 1 |
| Kou 2022 (12) | 1 |  | 1 |  | 1 |  | 1 |  | 1 |  | 1 |  | 1 |  | 1 |  | 1 |  | 1 |  |  |
| Tessitore 2012 (13) | 1 |  | 1 |  | 1 |  | 1 |  | 1 |  | 1 |  |  | 1 |  | 1 | 1 |  |  |  | 1 |
| Mi 2017 (14) | 1 |  | 1 |  | 1 |  | 1 |  | 1 |  | 1 |  | 1 |  | 1 |  | 1 |  |  | 1 |  |
| Lenka 2016(15) | 1 |  | 1 |  | 1 |  | 1 |  |  | 1 |  | 1 |  | 1 |  | 1 | 1 |  |  | 1 |  |
| Hu 2020 (16) | 1 |  | 1 |  |  | 1 | 1 |  | 1 |  | 1 |  | 1 |  | 1 |  | 1 |  |  |  | 1 |
| Bharti 2020 (17) |  | 1 | 1 |  | 1 |  | 1 |  | 1 |  | 1 |  | 1 |  | 1 |  | 1 |  | 1 |  |  |
| Wang 2022 (18) | 1 |  | 1 |  | 1 |  | 1 |  | 1 |  | 1 |  |  | 1 |  | 1 | 1 |  | 1 |  |  |
| Lench 2021 (19) | 1 |  | 1 |  | 1 |  | 1 |  | 1 |  | 1 |  | 1 |  |  | 1 | 1 |  | 1 |  |  |
| Lench 2025 (20) | 1 |  | 1 |  | 1 |  | 1 |  | 1 |  | 1 |  | 1 |  | 1 |  | 1 |  |  |  | 1 |
| Steidel 2021 (21) | 1 |  | 1 |  | 1 |  | 1 |  | 1 |  | 1 |  |  | 1 |  | 1 | 1 |  |  | 1 |  |
| Droby 2021(22) | 1 |  | 1 |  | 1 |  | 1 |  | 1 |  | 1 |  | 1 |  | 1 |  | 1 |  |  | 1 |  |
| Snijders 2011 (23) | 1 |  | 1 |  | 1 |  | 1 |  | 1 |  | 1 |  |  | 1 | 1 |  | 1 |  | 1 |  |  |
| Gilat 2015 (24) | 1 |  | 1 |  | 1 |  | 1 |  |  | 1 | 1 |  | 1 |  | 1 |  | 1 |  |  |  | 1 |
| Shine 2013 (25) | 1 |  | 1 |  | 1 |  | 1 |  | 1 |  | 1 |  | 1 |  | 1 |  | 1 |  |  |  | 1 |
| Xie 2025 (26) | 1 |  | 1 |  | 1 |  | 1 |  | 1 |  | 1 |  | 1 |  | 1 |  | 1 |  | 1 |  |  |
| Camastra 2025 (27) |  | 1 | 1 |  | 1 |  | 1 |  | 1 |  | 1 |  |  | 1 |  | 1 |  | 1 |  |  | 1 |
| Rubino 2014 (28) | 1 |  | 1 |  | 1 |  | 1 |  | 1 |  | 1 |  |  | 1 |  | 1 | 1 |  |  |  | 1 |
| Jha 2015 (29) | 1 |  | 1 |  | 1 |  | 1 |  | 1 |  |  | 1 |  | 1 |  | 1 |  | 1 |  | 1 |  |
| Tessitore 2012 (30) | 1 |  | 1 |  | 1 |  | 1 |  | 1 |  |  | 1 |  | 1 |  | 1 | 1 |  |  | 1 |  |
| Vaštík 2017 (31) | 1 |  | 1 |  | 1 |  | 1 |  | 1 |  | 1 |  | 1 |  | 1 |  |  | 1 |  |  | 1 |
| Kostic 2012 (32) |  | 1 | 1 |  | 1 |  | 1 |  | 1 |  | 1 |  |  | 1 |  | 1 | 1 |  | 1 |  |  |
| Tard 2015 (33) | 1 |  | 1 |  | 1 |  | 1 |  | 1 |  | 1 |  |  | 1 |  | 1 | 1 |  | 1 |  |  |

between neuroimaging results and FOG severity in PD freezers.

**Table S3. Characteristics of studies included in narrative synthesis**

| **Author Year** | **Imaging technique/ Task^¶^** | **Analysis type** | **Sample** | **Age** | **Sex M/F** | **HY** | **UP3** | **FOGQ^#^** | **Med** | **LED** | **Key brain regions in FR compared with noFR/HA, correlations with FOG outcomes in FR^¶^** |
| --- | --- | --- | --- | --- | --- | --- | --- | --- | --- | --- | --- |
| Shine  2013 (34) | task-fMRI/ VR | A priori ROI activation | 18 FR | 66.8 | 18/0 | 2.2 | 39.2 | 12.4 | Off | nr | FOG vs. walking: Increased in bilateral DLPFC and PPC; decreased in bilateral caudate, GPi, STN, and MLR. Correlations: FOG severity was inversely correlated with right DLPFC; positively with left MLR. |
| Shine  2013  (35) | task-fMRI  /VR | ICA, A priori ROI activation, FC | 10 FR 10 noFR | 67.1 66.3 | nr | 2.3 2.7 | 26.2 19.7 | 11.6 2.2 | On &  Off | 869  755 | *FR < noFR*: Motor and BG networks during high cognitive load, and in ventral attention network during Off state. *Correlations*: FOG frequency was associated with reduced FC of left and right cognitive control, and BG—bilateral cognitive control networks. |
| Lench  2024  (36) | rs-fMRI | Seed-voxel FC | 22 FRres 16 FRun  17 noFR | 65.1  69.8  68.6 | 15/7 12/4  14/3 | 2.1 2.4  2.0 | 16.7 19.3  18.6 | 20.6 21.3 / N | On | 1264  980 | *Dopa-responsive FR (FRres) > noFR*: FC between left STN and left lateral occipital cortex. *Dopa-unresponsive FR (FRun) vs. noFR*: FRun showed decreased FC between right STN and cerebellum; increased FC between left STN and left postcentral gyrus. |
| Matar  2019  (37) | task-fMRI/ VR | Whole-brain activation,  ROI-to-ROI | 19 FR | 65.3 | nr | 2. | 30.4 | 10.16 | On &  Off | 960 | FR (Off < On): Reduced activation in bilateral pre-SMA; decreased FC pre-SMA—STN during virtual doorway navigation. *Correlations*: Prolonged footstep latency was associated with reduced FC of pre-SMA—STN; positively with increased FC of bilateral STN. Rt=2; Lt=2 |
| Sarasso  2021 (38) | task-fMRI/  action observation | A priori ROI activation | 24 FR 18 HA | 66.5 65.5 | 17/7 9 /9 | 2.3 | 25.1 | 12.0 | Off | 954 | *FR < HA*: Right IFG, SMA, bilateral supramarginal during FOG-observation. *Interaction (group × condition)*: FR showed increased activation in left anterior medial PFC and reduced activation in dorsomedial PFC and bilateral hippocampus. *Correlations*: FOGQ was inversely related to left superior medial frontal gyrus during FOG-observation. |
| Brugger 2015 (39) | s-MRI | VBM | 18 FR 20 noFR | 61.1 61.5 | 6/12 15/5 | 2.3 2.1 | 29.3 22.8 | 11.7 2.2 | n/a | 1220  755 | *Correlations*: FOG severity was negatively correlated with GM volume in right MFG, superior/middle orbital gyri, SFG, MTG, parietal regions. In conjunction analysis, GM volume in right DLPFC was associated with both FOG and executive dysfunction. |
| Hvingelby 2025 (40) | PET | PCA, metabolic activity | 14 FR | 68.8 | 9/5 | nr | 35.9/m | 20.8 / N | On | nr | *Correlations*: ON-state FOG severity was negatively correlated with cholinergic terminal density in bilateral striatum (caudate and putamen), thalamus, hippocampus, amygdala, ACC, brainstem (MLR). |
| Gan  2023  (41) | rs-fMRI,  s-MRI | Group ICA, graph theory, VBM | 52 FR 73 noFR 38 HA | 62.7 61.7 61.9 | 28/24  47/26  25/13 | 2.3 2.2 | 33.0 26.6 | 12.1 1.0 | Off | 733  796 | *FR > noFR*: Dynamic FC of left precuneus—right caudate in State 2. *FR < noFR*: Variability in nodal efficiency of left precuneus. *Correlations*: FOGQ was positively correlated with mean dwell time and fractional windows of State 2. |
| Potvin-Desrochers 2023 (42) | rs-fMRI | Seed-based analysis | 15 FR  15 noFR | 69 64 | 7/8 9/6 | 2.5 2 | 37 26/ m | 12 /N | On & Off | 882 572 | *FR > noFR*: FC between BG/thalamus seeds and posterior parietal cortex, motor cortices, IFG in On-state. *FR < noFR*: FC between posterior cingulate/medial prefrontal cortex and BG/thalamus seeds in OFF-state. These L-DOPA-induced FC changes did not correlate with NFOGQ. |
| Fling  2014  (43) | rs-fMRI&  s-MRI&  d-MRI | ROI-ROI, Probabilistic Tractography | 7 FR  8 noFR 14 HA | 63.9 65.4 66.9 | PD14/1  HA5/9 | 2.6  1.9 | 36.3 29.6 | 18.1 0.4/ N | Off | 625.0 504.3 | *FR > noFR*: FC of SMA—bilateral MLR. FR < noFR: FC of SMA—right STN. HA > FR & noFR: Structural connectivity of right STN—SMA. Correlations: FC of SMA—MLR/CLR was positively correlated with FOG severity. |
| Sunwoo  2013 (44) | s-MRI | ROI-based volumetric | 16 FR,  30 noFR | 66.7 68.8 | 7/11  14/16 | nr | 30.7 18.7 | nr | n/a | 554.7 507.5 | FR < noFR: Reduced GM volume in the bilateral thalami, with no group difference in the substantia innominata. In PD, bilateral thalamic volume was positively correlated with visual recognition memory. |
| Pietracupa 2018 (45) | s-MRI&  d-MRI | Surface-based morphometry, DTI & Probabilistic Tractography | 21 FR 16 noFR 19 HA | 66.3 69.7 66.7 | 16/5 13/3 nr | 2  2.5 | 35.1  29.8 | 14.47 | Off | nr | *FR < HA* in GM: Decreased cortical thickness in bilateral mesial surface (frontal, parietal, occipital) and right dorsolateral prefrontal cortex. *FR vs HA* in WM: Widespread DTI abnormalities predominantly in the right hemisphere (superior and inferior longitudinal fasciculi, uncinate, cingulum) and forceps minor. *FR < noFR* in GM: Decreased surface area in the right parietal cortex (postcentral and supramarginal gyri, superior parietal lobule). |
| Guo  2026 (46) | rs-fMRI | ALFF, ReHo, Degree Centrality | 28 FR 30 noFR 33 HA | 63^†^ 64 64 | 15/13 16/14  13/20 | 2^†^  2 | 30.9 30.5 | 11 0 | nr | 300 300 | *FR vs. noFR*: Left parahippocampal gyrus, right IFG, left superior occipital gyrus, right supramarginal, left cerebellum. Correlations: mean ALFF values in left parahippocampal gyrus negatively correlated with FOGQ. |
| Herman  2014 (47) | s-MRI | Whole-brain VBM & ROI analysis | 30 FR 76 noFR | 65.1  64.9 | 26/4 55/21 | 3.18 2.33 | 38.4 45.9 | 15.23  /N | nr | 646.4 478.4 | FR < noFR (GM): Inferior parietal lobule, angular gyrus in matched groups. Correlations: NFOGQ was negatively correlated with bilateral caudate volume in entire cohort; Inferior parietal lobule, angular gyrus, caudate in matched group.  Inferior parietal lobule atrophy correlated with poorer cognitive function and slower dual task gait speed. |
| Peterson  2021 (48) | s-MRI | Probabilistic Tractography | 21 FR 18 noFR 19 HA | 68  67 69 | 18/3 15/3 16/3 | 2.2^‡^ 2.1 | 43 34 /  m | 14.1 / N | Off | nr | *HA > PD*: FA, FR vs. noFR: no differences in FA of the response inhibition network (connections of right inferior frontal cortex, —pre-SMA, —subthalamic nucleus). Correlation in FR: FA in the right preSMA and right STN tracts was positively correlated with FOG severity. |
| Jin C 2023  (49) | rs-fMRI | Short- & long-range FC density | 23 FR 36 noFR 22 HA | 66.4 63.5 62.5 | 12/11 18/18 9/13 | 2.24 2.31 | 23.4 22.4 | 9.28  1.34 | Off | 427.3 367.6 | *FR > noFR*: short-range FC density in left fusiform gyrus, temporal areas (right MTG, bilateral ITG), FR < noFR: long-range FC density in right frontal areas (SFG, MFG, IFG), ACC. Correlations: FOGQ was positively correlated with short-range FC density in right MTG/ITG; negatively with long-range FC density in right MFG. |
| Jin C 2022 (50) | s-MRI&  d-MRI | Graph Theory & Network-Based Statistics | 21 FR 34 noFR 23 HA | 65.5 64.7  64.2 | 10/11 17/17 9/14 | 2.43 2.13 | 23.4 22.3 | 9.39 1.24 | Off | nr | *FR vs. HA*: Decreased normalized clustering coefficient, small-worldness, clustering coefficient; increased local efficiency. *FR < noFR*: Betweenness Centrality in caudate, structural connections in frontal, temporal, parietal lobes. Correlations: Betweenness centrality in left SMA, right caudate was negatively correlated with FOGQ. |
| Lima-Pardini 2020 (51) | task-fMRI  /APAs | Whole-brain/ ROI activation, ROI-ROI & Graph Theory | 20 FR 18 noFR | 57.6 67.4 | 14/6 13/5 | nr | 33.5  28.5 | 18.95/ N | On | 753.8 437.5 | *FR > noFR*: Activation in right anterior insula and SMA during APAs. Local efficiency and clustering coefficient in SMA and left anterior insula. FR < noFR: FC between bilateral insulae, which negatively correlated with NFOGQ. Correlation in FR: SMA betweenness centrality positively correlated with FOG severity. |
| Moreira-Neto  2022 (52) | task-fMRI  /leg lifting | A priori ROI activation | 34 FR | 63.7 | 26/8 | 3.2 | 49.9 | 22.2 | On | 802.6 | Right MLR activity was a common neural substrate explaining the variance of all three variables. Right DLPFC activity explained variance in FOG severity and gait automaticity, but not APA amplitude. |
| Huang  2021  (53) | task-fMRI/ imagery | Whole-brain & ROI activation | 20 FR 14 noFR 15 HA | 66.0 69.8 63.4 | 12/8 8/6 5/10 | 3.1 2.2 | 30.4 24.4 | 20.7/ N | Off | 775.6 580.6 | *FR > noFR & HA*: Activation in CLR, MLR, SLR during normal turning imagery. FR > noFR: Activation in superior occipital, left precentral gyrus, and right postcentral gyrus in FOG turning vs. FOG straight walking. |
| Silva-Batista  2024 (54) | task-fMRI/  leg lifting | ROI analysis | 34 FR | 63.7 | 26/8 | 3.2 | 49.9 | 22.2 | On | 802.6 | Decreased activation of MLR and reduced APA amplitude together explained 45% of the variance in the loss of presynaptic inhibition. |
| Youn  2015 (55) | s-MRI | Whole-brain VBM & ROI based DTI | 19 FR 23 noFR 33 HA | 71.1 67.7 69.6 | 11/8 14/9 19/14 | 2.3 2.2 | 21.8 18.9 | 13.1 | n/a | 853.2 792.3 | *FR > noFR*: Mean diffusivity in BG, thalamus, cerebellum; Mean diffusivity in bilateral PPN. FR < noFR: FA in bilateral PPN. Correlation in FR: FA value in right PPN was correlated with FOG severity. |
| Li  2020 (56) | s-MRI | Cortical thickness, volume-based | 24 FR 37 noFR 24 HA | 65.5 64.1 62.5 | 13/11 19/18 9/15 | 2.5 2.07 | 29.3  28.0 | 8.4 1.4 | n/a | 592  310 | *FR & noFR < HA*: Cortical thickness in the right MTG. *Correlations*: Right MTG thickness was negatively correlated with UPDRS2 and showed a borderline significant correlation with FOGQ. |
| Peterson  2015 (57) | s-MRI | Probabilistic Tractography | 13 FR 12 noFR | 66.2 64.9 | nr | 2.62 1.96 | 36.8 29.2 | nr/ N | n/a | nr | *FR > noFR*: Asymmetry in PPN structural connectivity (higher PPN laterality index). Correlations: dual task interference was strongly correlated with PPN laterality, Go-NoGo target accuracy, and simple reaction time. |
| Wang L 2023 (58) | rs-fMRI& s-MRI | Graph Theory, ROI-ROI | 30 FR 40 noFR 25 HA | 62.3 61.8 60.4 | 17/13 25/15 17/8 | 2.40 2.24 | 33.5 29.8 | 12.7 | Off | 645.3 686.5 | *FR > noFR*: Functional local efficiency. *FR < noFR*: structural global and local efficiency; Nodal efficiency in left SMA, gyrus rectus, middle cingulate cortex. *FR > HA*: Path length. FR < HA: Global efficiency. No differences in structural-functional coupling. Correlation in FR, reduced structural global and nodal efficiency correlated with FOGQ. |
| Maidan  2019 (59) | rs-fMRI | Graph Theory | 26 FR 11 noFR 20 HA | 73.9 74.3 69.6 | 15/11 7/4 10/10 | nr | 21.0  33.58 | 18 /N | On | 1425 524 | *FR > HA*: Local efficiency in dorsal attention network (DAN). FR < HA: Betweenness centrality in DAN. FR < noFR & HA: Global efficiency in dorsal attention network. FR < noFR: Global efficiency in right MFG. Correlation in FR: A trend towards a negative correlation between dorsal attention network global efficiency and NFOGQ. |
| Myers  2017 (60) | s-MRI | Cerebellar GM volumetry | 25 FR 38 noFR | 66.7 63.9 | 14/11 20/18 | 2.2^‡^ 2.1 | nr | 9.8 /N | n/a | nr | *FR vs. noFR*: No differences in cerebellar GM volumes (either total or individual lobules). Correlations: While smaller cerebellar volumes were associated with better executive function in noFR, this association was not observed in FR. |
| Gilat  2018 (61) | rs-MRI | ROI-ROI | 19 FR 21 noFR | 68.4 66.9 | nr | 2^†^ 2 | 42.7  36.1 | nr/N | Off | 820 460 | *FR > noFR*: FC of right amygdala—putamen, anti-coupling FPN—left amygdala. FR < noFR: Anti-coupling FPN**—**right putamen. Correlations: Worse FOG severity was associated with increased FCs of left amygdala—putamen, anti-coupling amygdala—FPN regions; greater anti-coupling FPN—bilateral amygdala was related to fear of falling. |
| D’Cruz  2021 (62) | rs-fMRI&  s-MRI | Vertex-based shape analysis & ROI-ROI | 36 noncon 9 convert 12 FR | 59  57 69 | 29/7 5/4 11/1 | HY1/2/3 9/21/6  0/7/1  0/9/2 | 23.5 30  39/ m | nr/N | Off | 412.5 814 639 | *Convert > noncon*: Longitudinally, local volume in left thalamus, brainstem; Thalamo-cortical functional coupling with limbic/cognitive regions prior to conversion, with a marked reduction in coupling with left DLPFC, right medial prefrontal cortex, left SPG. Correlations: At study entry, local thalamic inflations were positively associated with larger medial thalamic sub-nuclei volumes and better executive function. |
| Onder  2023 (63) | d-MRI | microstructure | 18 PDFR 13 PDnoFR 6 PSPFR 5 PSPnoFR | 69.6 68.6 67.6 67.6 | 16/2 7/6 6/0  3/2 | nr | 16.3 12.6 23.1 25.0 | PD 19.2 PSP 20.8 | n/a | 1190 1053 1190 1053 | *FR < noFR* in PD: Microstructural disturbances (integrity) in bilateral SFG, bilateral fastigial nucleus of cerebellum, left pre-SMA. PSP FR showed disturbances in left pre-SMA, and correlations between FOG severity and right STN and left PPN. |
| Wróbel  2025 (64) | s-MRI,  d-MRI | Cortical Thickness, microstructure | 10 FR 19 noFR | 64.2 64.1 | 7/3 11/8 | 2.7 2.3 | 39.3 32.5 | 7.22/  Ziegler Score | n/a | 1497 1251 | Lower right SMA FA correlated with better quantitative gait in PD, particularly during dual tasking; SMA FA did not differ between FR and noFR, nor did it correlate with clinical FOG or postural instability and gait difficulty scores. Cortical thickness showed no correlation with gait parameters in either analysis. |
| Ehgoetz Martens 2018 (65) | task-fMRI/  VR | ROI-ROI | 41 FR | 66.5 68.6 | 32/9 | nr | 33.9 | 2.5/FOGQ item3 | Off | nr | Functional decoupling cognitive control/motor networks—striatum; increased coupling limbic network—striatum (FOG vs. normal foot tapping). Correlations: Worse FOG severity was related to increased hyper-connectivity of cognitive—limbic networks. |
| Hoorn 2014 (66) | task-fMRI/ optic flow | Whole-brain activation, Seed-based | 15 FR 7 noFR 15 HA | 60.9 62.1 60.5 | 9/6 3/4 9/6 | 2.5^†^  2.5 | 32.7 21.6 | 10.1 1.2 | Off | 871  609 | PD < HA: Activation in pre-SMA and dorsal occipito-parietal regions during wide-field optic flow and its interruption. FR < noFR: FC between right visual motion area V5, pre-SMA, cerebellum. |
| Potvin-Desrochers 2019 (67) | rs-fMRI | Seed-based | 14 FR 13 noFR | 67.7 65.8 | 10/4  8/5 | 2.5 2.1 | 39  33 | 14/N | On | 874  677 | FR > noFR: FCs of bilateral thalamus—visual areas, bilateral external globus pallidus—visual areas. FC of left putamen—both retrosplenial cortex and cerebellum. Correlations: These FCs did not correlate with FOG severity or PD motor severity. |
| Galléa  2021 (68) | rs-fMRI | Seed-based | 17 FR 8 noFR 25 HA | 60.9 60.7 59.8 | 12/5 6/2 13/2 | 2.1 1.75 | 31.7 23.8 | 4.3 at follow-up | Off | 752  658 | FR > noFR: Longer baseline antisaccade latency, predicting FOG onset within 5 yrs. Correlations: In HA, stronger MLR connectivity with the left frontal eye field and right preSMA was associated with shorter antisaccade latency, whereas this association was reversed in PD. |
| Li N  2022  (69) | rs-fMRI | Graph Theory | 20 convert 20 noncon 20 HA | 54.7 54.5 53.3 | 10/10 10/10 10/10 | 1.8 1.7 | 20.0  20.0 | nr/ N | Off | 487.5 482.1 | *Convert < noncon*: Nodal centrality in left MFG at baseline. *Convert vs. HA*: Trends of altered centrality in visual and cerebellar regions. *Convert vs. noncon*: Specific disruption in executive network (left MFG) was the key differentiator. |
| Peterson  2014 (70) | task-fMRI/  imagery | ROI-based | 9 FR 9 noFR | 66.6 62.7 | 5/4 7/2 | 2.5 2.22 | 36.1 27.7 | 13.0 /N | Off | nr | FR < noFR: Activation in right GP during imagined gait (both simple and complex), CLR during imagined standing. |
| Ruan  2020  (71) | rs-fMRI | Graph Theory | 23 FR 33 noFR 24 HA | 65.0 63.8 62.3 | 11/12  18/15  12/12 | 2.45 2.09 | 28.8 27.9 | 9.59 1.60 | On | 494.5 316.6 | FR < noFR: Nodal local efficiency in right IFG opercular part and increased betweenness centrality in left hippocampus. Correlations: Local efficiency disruptions in right temporal pole (STG) were positively correlated with FOGQ. |
| Li N 2021  (72) | fMRI | Graph Theory | 37 FR 38 noFR | 54.8 55.1 | 19/18 23/15 | HY2/2.5/3  3/13/21  5/18/15 | 34.5  30.0 | 18.9/N | Off | 707.1 609.9 | FR < noFR: Small-worldness, centrality in frontoparietal regions, cerebellum. FR > noFR: Nodal centrality in sensorimotor (SMA), visual, subcortical (caudate, thalamus), limbic (hippocampus, amygdala) networks. Correlations: Centrality in right hippocampus was positively related to NFOGQ. |
| Zhou W 2023  (73) | d-MRI | Fixel-based, tractography | 19 FR 19 noFR 21 HA | 68.0 67.6 67.6 | 14/5 13/6 14/7 | nr | 39.1 35.2/  m | 1.21/  mUP3.11 | n/a | nr | *FR > noFR*: Fiber cross-section in corpus callosum (CC), fornix, ILF, striato-/thalamo-premotor tracts, superior thalamic radiation (STR); Fiber density and cross-section in STR. *FR < noFR*: Fiber density in CC and ILF. Correlations: ILF with motor severity, cognition, memory; CC with anxiety. |
| Zhang Y 2024  (74) | s-MRI | Gray matter & Neuromelanin (NM) | 25 FR 22 noFR 30 HA | 63.0  60.6 59.5 | 15/10  16/6 16/14 | 2.7 2.3 | 48.3  41.5 | 16.7  3.7 | n/a | nr | *FR > HA*: Iron deposition in the bilateral caudal substantia nigra. FR < noFR & HA: NMvolume-substantia nigra pars compacta (SNpc). *FR & noFR < HA*: Bilateral caudate and putamen volumes and decreased NMRC-SNpc. Correlations (multiple regression): FOG severity was positively predicted by left caudal SN iron content and left caudate volume, and negatively predicted by NMvolume-SNpc. |
| Fling  2013  (75) | d-MRI | Probabilistic Tractography | 14 FR 12 noFR 15 HA | 67.1 65.5 66.7 | 13/1 9/3 10/5 | 2.7  2.0 | 37.1 29.3 5.7 | 17.3 0.3 /N | n/a | 646 485 | FR < HA & noFR: Right PPN tract volume/connectivity, especially to thalamus, cerebellar locomotor region, frontal/prefrontal cortical regions. *FR > HA & noFR*: Left-lateralization of PPN tract volume, which in FR was related to poorer inhibition-related tasks. |
| Gan C 2023  (76) | s-MRI | VBM & Surface-Based Morphometry | 20 FR 20 noFR 21 HA | 62.6 60.6 61.8 | 8/12  14/6  14/7 | 2.53 2.15 | 31.2 23.0 | 10.76 | n/a | 649.4 533.9 | *FR < noFR & HA*: Atrophy in Cholinergic nucleus 4 (Ch4) of basal forebrain; Cortical gyrification in left entorhinal cortex and parahippocampal gyrus. In FR, Ch4 volume was negatively correlated with FOGQ. |
| Yang  2025  (77) | s-MRI | ROI analysis, Neuromelanin | 22 FR 24 noFR 13 HA | 70.6 63.8 64.8 | 13/9 14/10 8/5 | nr | 76.4 42.8 | 14  2 | n/a | nr | *FR < noFR; PD < HA*: Reduced contrast-to-noise ratio in the left locus coeruleus. Multiple linear regression showed that FOG was an independent factor associated with lower left locus coeruleus contrast-to-noise ratio. |
| Gan  2022 (78) | s-MRI | VBM | 25 FR 53 noFR 29 HA | 64.5 62.4 52.3 | 11/14 28/25 13/16 | nr | 45.7 38.5 | nr | n/a | 1094 927 | *FR < noFR & HA*: GM density in right superior occipital gyrus. FC of right superior occipital gyrus—right paracentral lobule was reduced in FR, which was negatively correlated with FOGQ. |
| Bharti  2019  (79) | rs-fMRI&  s-MRI &  d-MRI | Seed-based, Tract-based spatial statistics, VBM | 15 FR 16 noFR 16 HA | 71.0 66.3 66.7 | 12/3 13/3 6/10 | 2.3 2 | 36.2 29 | nr | Off | nr | *FR > HA*: FCs of CLR—cerebellar, —parieto-occipital cortex. FC of fastigial nucleus—cerebellar/  posterior cortical areas. *FR < noFR*: FCs of dentate nucleus—brainstem, —right BG, —frontal/  parieto-occipital cortices. Structurally, *FR > HA/noFR:* FA in superior cerebellar peduncle/middle cerebellar peduncle; FR *vs noFR:* Mean diffusivity in superior cerebellar peduncle. *Correlations*: Increased FC CLR-cerebellar was positively related to FOGQ; structural abnormalities with worse FOG severity. |
| Quek  2024  (80) | rs-fMRI | ROI Analysis | 23 FR | 77 | 19/4 | 2.9 | 43.7 | 11.9 | On & Off | 836.7 | OFF state showed increased coupling between right hippocampus and right caudate compared to ON. Anxiety levels correlated with changes in connectivity between left amygdala and left prefrontal cortex when moving from OFF to ON states |
| Lv  2022  (81) | rs-fMRI | Seed-based  (MRI cohort): | 20 FR 21 noFR 22 HA | 58.9 60.1 57.9 | 8/12 9/12 12/10 | 2.5 2.5 | 35 30 | nr | Off | nr | *FR < noFR & HA*: FC between dorsal raphe nucleus and cortical structures (including SMA, left SFG, left median cingulate cortex). No correlation between dorsal raphe nucleus connectivity and FOG severity. |
| Vervoort  2016  (82) | rs-fMRI | ROI-ROI | 13 FR 60 noFR 20 HA | 65.8 58.2 57.7 | 1/12  31/29 14/6 | 2.2 2.0 | 38.3 25.9 | 15.8/ N | Off | 604.8 409.7 | *FR < noFR*: FC of left caudate—right STG/left cerebellum. FCs of right caudate—bilateral dorsal putamen/left GP/bilateral STG. *FR > noFR*: FC of left dorsal putamen—right precuneus. Correlations: Worse DT parameters correlated with decreased FC of left caudate—STG, and right caudate—left GP; and with increased FC of left dorsal putamen—right precuneus. |
| Jin C  2021  (83) | rs-fMRI | Graph Theory | 24 FR 37 noFR 22 HA | 65.5 64.1 62.5 | 13/11 19/18 9/13 | nr | 22.4 21.3 | 9.25 1.54 | Off | nr | FR < noFR: Degree centrality in right inferior temporal gyrus and left MFG. Correlations: Worse FOGQ correlated with decreased degree centrality in right ITG. |
| Huang  2024  (84) | rs-fMRI | ROI-ROI, Graph Theory | 10 FR 8 noFR 10 HA | 56.9  56.4 56.0 | 7/3  3/5  6/4 | 3.3 2.9 | 37.4  36.0 | 15.5 4.8 | Off | 117 1129 | *FR > noFR*: FC of left IFG network—bilateral postcentral gyri. *FR < noFR*: 10 FCs including vermis 12–left temporal occipital fusiform cortex, right caudate–left frontal/temporal regions*. noFR < HA*: Clustering coefficient in right posterior STG. *FR < noFR & HA*: FC of right caudate—left anterior ITG. Correlations: Reduced vermis 12–left temporal occipital fusiform FC was related to FOGQ and UPDRS; Increased left IFG–right postcentral FC with UPDRS3. |
| Canu  2015  (85) | rs-fMRI,  s-MRI,  d-MRI (1.5T) | VBM, DTI (Tract-based spatial statistics & probabilistic tractography), Independent component analysis | 28 FR 25 noFR 30 HA  (1.5T) see Supplementary Material | 65.4 64.4 63.9 | 22/6 15/10 14/16 | 2.7 2.5 | 45.1 41.9 | 8.0 2.7 | On | 986.6 798.4 | *FR > noFR*: Axial diffusivity (axD) in the genu/body of the corpus callosum and WM underneath right parietal cortex. FR < HA: FC within sensorimotor, default mode network, visual associative networks; widespread WM damage (Mean diffusivity↑/FA↓) involving pedunculopontine tract, corpus callosum, corticospinal tract, cingulum, superior longitudinal fasciculus, and cortical WM regions. Correlations: greater right PPT axD was associated with worse UP3. |
| Vastík  2016 (86) | task-fMRI/  Imagery (1.5T) | Whole-brain activation | 11 FR 10 noFR | 71.2 69.7 | nr | nr | nr | nr | Off | nr | Baseline (W0) *FR < noFR*: Right temporo-occipital cortex, right occipital pole. Correlations: FOG positively correlated with activation in left superior parietal lobule, medial occipital cortex, right lateral occipital cortex, lateral cerebellum; TUG positively related to right superior parietal lobule, intraparietal sulcus, medFC, right putamen, caudate, bilateral temporal poles, pons; negatively with bilateral mediodorsal pre/postcentral gyri |
| Saçmaci 2021 (87) | s-MRI (1.5T) | Facial nerve thickness | 35 FR 35 HA | 70.2 66.0 | 23/12 25/9 | 3 | 22.8 | 8 | n/a | 409.3 | *PD < HA*: Right facial nerve thickness (proximal and distal), facial nerve-to-internal acoustic channel (IAC) ratio. Correlations: Worse FOG severity was related to facial nerve-to-IAC ratio. |
| Sarasso  2022  (88) | rs-fMRI  s-MRI (1.5T) | Cortical thickness, BG volumetry, Graph Theory | 11 noncon 11 covert 30 FR 30 HA | 63.8 61.9 63.8 63.7 | 6/5  7/4  22/8 21/9 | 1.91 2.54 2.63 | 32.8 43.6 47.1 | 9.40 | On | 690.5 893.6 955.8 | *FR < HA*: Cortical thickness in bilateral superior temporal sulci, left sensorimotor cortex, right supramarginal area; Volume in bilateral caudate, left putamen. *Convert < HA*: Left sensorimotor cortical thickness, right caudate volume. Correlations: Baseline left precentral atrophy was related to worsening executive functions over 2 yrs. |
| Youn  2012  (89) | d-MRI (1.5T) | DTI | 14 FR 26 noFR | 80.6 79.4 | 8/6 15/11 | nr | nr | 6.07 | n/a | nr | FR < noFR: FA values in PPN, bilateral SPG, right orbitofrontal area, left SMA. Tractography also showed decreased fiber bundle volume connecting PPN to multiple cortical areas. Correlations: No correlation between FOG and FA in any of the regions. |
| Trufanov  2016  (90) | d-MRI (1.5T) | Diffusion-tensor tractography | 31 FR 47 noFR | 62.7 61.1 | nr | 3  3 | nr | 30.7/ Gait & Balance scale | n/a | nr | *FR vs. noFR*: Bilateral anterior thalamic radiations (ATR) abnormalities, left cingulum bundle changes, left temporal and right superior longitudinal fasciculus abnormalities, reduced right ILF tract length, and increased diffusivity in right corticospinal tract. *Correlations*: worse Gait & Balance scale correlated with abnormalities in right ATR, left cingulum bundle, right ILF. |
| Tard  2025 (91) | PET | A priori ROI metabolic connectivity | 12 FR 14 noFR | 62 62.3 | 3/8  4/10 | nr | 33.5 26.9 | 12.1 0.84 | Off | nr | *FR < noFR*: Disrupted thalamo-cortical and cerebello-cortical loops, temporo-parietal networks, and frontal networks at rest. During actual gait, connectivity from cerebellar regions remained lower. *FR > noFR*: Connectivity from occipital and parietal regions towards internal frontal/orbitofrontal regions during actual gait. |
| Zhou  2019  (92) | Hybrid PET-CT | Volume of interest analysis | 20 FR 147 noFR | 66.9 56.3 | 11/9 87/60 | 2.25 1.99 | 43.4 37.3 | nr | nr | 704.1 535.6 | *FR < noFR*: Dopamine levels in putamen, specifically anterior and dorsal putamen. Glucose metabolism (18F-FDG uptake) in left primary visual cortex. *FR > noFR*: Glucose metabolism in caudate nucleus and lentiform nucleus. |
| Bartels  2006 (93) | PET | ROI, Voxel-based analysis | 10 FR  7 noFR | nr | nr | 2.6 2.3 | 12.8 9.4 | 15.1 | On | nr | *FR < noFR*: Glucose in bilateral caudate, parietal regions, left superior premotor; Dopamine in right putamen, bilateral caudate. *FR > noFR*: Glucose in bilateral posterior putamen. |
| Mitchell 2019  (94) | PET | ROI analysis, Whole-brain voxel wise analysis | 9 FR 9 noFR | 68 65 | 5/4 8/1 | 3 2 | 48 41 | 13/ N | Off | 893 751 | *FR > noFR:* Right Crus II, SMA, left superior medial gyrus, posterior-medial frontal gyrus, middle/superior temporal gyri; Thalamus, precentral gyrus, right frontal gyri (less deactivation). *Correlations*: Increased right DLPFC activity during steering correlated with reduced stride length, but not with FOG severity. |
| McKay  2023  (95) | PET | ROI analysis | 16 noFR 10 FRresp 21 FRunresp 5 PP-FOG | 67.3 68.5 68.6 65.6 | 11/5 9/1 19/2 2/3 | nr | 31.1 30.6 34.3 39.4 | 0.0 16.7 21.2  17.8/N | On | 833 1149 1531 1258 | *FRunresp < noFR*: Norepinephrine transporter binding in frontal cortex, temporal lobe, locus coeruleus, and bilateral thalamus (most prominently in right thalamus). FOG-Resp vs. primary progressive (PP)-FOG: similar to noFR. Correlations in FR: In OFF-FOG, lower right thalamic Norepinephrine transporter binding was correlated with higher NFOGQ. |
| Imamura 2012  (96) | SPECT | Regional cerebral blood flow | 21 FR 34 noFR | 71.5 69.1 | 13/8 22/12 | 2–4  1–5  (range) | 26.1  20.9 | 2.6 / UP2item | Off | 323.8 276.5 | *FR < noFR*: Regional cerebral blood flow in bilateral BA10/11 and left BA32, especially BA11. Correlations in FR: Higher Z-scores in bilateral BA10/11 and left BA32 were correlated with higher FOG score, especially in bilateral BA11. |
| Matsui  2005  (97) | SPECT | Whole-brain brain perfusion | 24 FR 31 noFR | 66.5 70.3 | 11/13  9/22 | 3.0 3.1 | 32.8 30.6 | 3.5/ FOG3 | nr | 317 265 | *FR < noFR*: Perfusion in bilateral BA11 (orbitofrontal cortex); at a more liberal threshold, reduced perfusion in medial frontal area. Correlations: Lower BA11 perfusion was correlated with higher FOG score; with disease duration, but not with UPDRS, H&Y stage, age, or MMSE. |
| Miranda-Domínguez 2020 (98) | rs-fMRI | ROI-ROI,  Seed-based (whole cohort) | 28 FR 36 noFR 39 HA | 68.9 69.2 69.5 | nr | nr | 45.9  36.5 /m | nr | Off | 1405 683.9 | *FR > noFR & HA*: FC of left globus pallidus—left somatosensory cortex. *FR < noFR < HA*: FC of default mode network—left auditory/vestibular network. Correlations: FC of left GP—left motor cortex was positively related to objective FOG severity; negatively with foot pitch angle. |
| Wang X 2025 (99) | s-MRI | Parcellation, Principal component analysis | 153 FR 163 noFR | 69 65 | 87/66 95/68 | 3 3 | 46  36/m | 3^†^ | n/a | 500 300 | *FR < noFR*: Volume of the ventral diencephalon, pallidum, and thalamus. *Correlations*: In cohort 1, ventral diencephalon volume was not robustly correlated with FOG severity after adjustment. |
| Lench  2022 (100) | s-MRI,  d-MRI | Diffusion kurtosis, Tractography | 21 FRresp 15 FRunresp | 65.0 70.9 | 14/7  10/5 | nr | 10.2 6.0 | 18.1 22.5/N | n/a | 1213 1083 | *FRunresp < FRresp*: FA in bilateral GPi, mean kurtosis in right GPi, right GPi–MLR tract. FRunresp > FRresp: Radial diffusivity in right GPi, Mean diffusivity in right GPi–MLR tract. Correlations: Lower right GPi mean kurtosis was related to poorer FOG dopa-response. |
| Naduthota 2017 (101) | s-MRI | Iron accumulation | 17 FR 17 noFR 34 HA | 54.5 54.8 55.3 | 12/5 12/5 24/10 | HY1/2/3 0/11/6 6/9/2 | 22.2 16.9 (On) | 11.9 1.8 | n/a | 578 565 | *FR > noFR*: Iron accumulation (R2*) in SNc, especially caudal SNc. FR < noFR: None reported. Correlations in FR: Higher caudal SNc R2* was correlated with higher FOG score. |
| Roytman 2023 (102) | PET | Voxel-wise, Volume of interest-based | 16 FR 59 noFR | 67.5 65.0 | 11/5 45/14 | nr | 35.2 25.5/m | nr | On | 685.0 450.0 | FR vs. noFR: No differences in right cortical cholinergic. Correlations: Greater postural instability during proprioceptive cue loss was correlated with reduced cholinergic integrity in right cerebral hemisphere. High anti-cholinergic burden predicted postural control impairment, dependent on right hemispheric cortical cholinergic integrity. |
| Taylor  2022  (103) | task-MRI/ VR | A priori ROI-based dynamic FC | 26 FR | 67.9 | 19/7 | nr | 37.4 | 2.43/  FOG3 | Off | nr | *Threatening < non-threatening*: Limbic within-network connectivity; coupling within distinct cognitive regions with greater task-related increase in % time frozen. *Threatening > non-threatening*: Increased network integration across bilateral motor, limbic, and cognitive control networks. |
| Maillet 2015 (104) | PET/ imagery | Whole-brain voxel-based | 8 FR | 63.3 | 4/4 | 3.4 Off 2.3 On | 37.8 14.9 | 18 | On&  Off | 916 | During gait motor imagery, OFF state showed greater premotor-parietal and pontomesencephalic activity, whereas ON state showed greater motor, putaminal, thalamic, and cerebellar activation and reduced premotor-parietal/brainstem involvement. |
| Chou 2025  (105) | PET | Whole-brain voxel-based | 20 FRresp 16 FRunresp | 69.3 71.0 | 15/5  16/0 | 2.7 2.9 | 48.8 45.8 | Video-based FOG | nr | 926.6 778.4 | *FRunresp < FRresp*: greater cholinergic terminal loss in bilateral insula/hippocampi/  fimbria/metathalamus, plus left putamen/posterior cingulate/mid-temporal and right mid-frontal/anterior ventral thalamic regions. Correlations: lower cholinergic integrity was associated with poorer levodopa responsiveness of FoG. |
| Raghu 2021  (106) | s-MRI,  d-MRI | Probabilistic Tractography, cortical thickness | 8 FR | 67.4 | 8/0 | nr | nr | 16.3 | n/a | nr | Better outcome was associated with higher volume of activated tissue (VAT) connectivity to precentral gyrus and postcentral BA1/2, and lower activated tissue connectivity to superior cerebellar peduncle; VAT-precentral connectivity also related to FOGQ improvement. Greater baseline GFQ severity was related to lower cortical thickness in left parietal lobe and bilateral premotor cortices. |
| Hall  2018  (107) | d-MRI | Structural connectome, Graph Theory | 26 FR 24 noFR | 64.2 66.9 | nr | 2 1.8 | 25.5  23.3 | nr | n/a | 657  527 | *FR < noFR*: Participation coefficients in right caudate, thalamus, hippocampus, frontoparietal cortices. Module degree z-scores in prefrontal, sensorimotor, visual association cortices. *FR >* *noFR*: Module degree z-scores in DLPFC, frontal eye fields, somatosensory/visual association, inferior temporal region. |
| Vercruysse 2015  (108) | d-MRI | Tract-based spatial statistics, Probabilistic tractography | 11 FR 15 noFR 15 HA | 68.6 67.6 68.1 | 8/3  11/4  11/4 | 3  2.5 | 36.6 32.5 | 15^†^ | n/a | 704  461 | *FR < noFR*: FA in bilateral cerebellum, left superior longitudinal fasciculus, left middle cerebellar peduncle-PPN tract; Connections between left caudate and anterior cingulate/orbitofrontal cortices. *FR > noFR*: Mean diffusivity in right internal capsule, anterior corona radiata, SFG, left anterior thalamic radiation, cerebellum. |

These studies were included in the narrative synthesis only and were excluded from the ALE meta-analysis.

Data are shown as means unless otherwise indicated († median); ‡ values were calculated from category distributions. ¶ If applicable. # Scores represent the Freezing of Gait Questionnaire (FOGQ); 'N' indicates the New Freezing of Gait Questionnaire, and 'm' indicates the Movement Disorder Society-Sponsored Revision of the Unified Parkinson's Disease Rating Scale Part 3.

Abbreviations: UP3 = Unified Parkinson's Disease Rating Scale Part 3, s-MRI = structural MRI, d-MRI = diffusion MRI, FR = freezers, noFR = non-freezers, HA = healthy older adults, n/a = not applicable, nr = not reported, Med = medication status during scan, LED = levodopa equivalent daily dose, FC = functional connectivity, VBM = voxel-based morphometry, VR = virtual gait task, GM = gray matter, WM = white matter, ILF = inferior longitudinal fasciculus, BA = Brodmann area, FA = fractional anisotropy, PSP = progressive supranuclear palsy, STG = superior temporal gyrus, IFG = inferior frontal gyrus, MLR = mesencephalic locomotor region, PPN = pedunculopontine nucleus, MFG = middle frontal gyrus, ITG = inferior temporal gyrus, BG = basal ganglia, SMA = supplementary motor area, STN = subthalamic nucleus, SFG = superior frontal gyrus, FOGQ = Freezing of Gait Questionnaire (FOG3 = Item3 of FOGQ), NFOGQ = New Freezing of Gait Questionnaire, MTG = middle temporal gyrus, CLR = cerebellar locomotor region, DLPFC = dorsolateral prefrontal cortex, GP = globus pallidus, GPi = internal globus pallidus.

**References for supplementary material**

1.	Li Y, Ruan X, Li E, Zhang G, Liu Y, Du Y, et al. Aberrant Advanced Cognitive and Attention-Related Brain Networks in Parkinson's Disease with Freezing of Gait. Neural Plast. 2020;2020:8891458.

2.	Li X, Hou M, Qu Y, Huo Y, Liu S, Ma M, et al. Gait dynamics and brain function abnormalities in Parkinson's disease with freezing of gait: a clinical study using resting-state fMRI and wearable devices. Front Neurosci. 2025;19:1560333.

3.	Piramide N, Agosta F, Sarasso E, Canu E, Volonte MA, Filippi M. Brain activity during lower limb movements in Parkinson's disease patients with and without freezing of gait. J Neurol. 2020;267(4):1116–26.

4.	Yu Q, Li Q, Fang W, Wang Y, Zhu Y, Wang J, et al. Disorganized resting-state functional connectivity between the dorsal attention network and intrinsic networks in Parkinson's disease with freezing of gait. Eur J Neurosci. 2021;54(7):6633–45.

5.	Taniguchi S, Kajiyama Y, Kochiyama T, Revankar G, Ogawa K, Shirahata E, et al. New Insights into Freezing of Gait in Parkinson's Disease from Spectral Dynamic Causal Modeling. Movement Disorders. 2024;39(11):1982–92.

6.	Lench DH, Embry A, Hydar A, Hanlon CA, Revuelta G. Increased on-state cortico-mesencephalic functional connectivity in Parkinson disease with freezing of gait. Parkinsonism Relat Disord. 2020;72:31–6.

7.	Guo M, Ren Y, Yu H, Yang H, Cao C, Li Y, et al. Alterations in Degree Centrality and Functional Connectivity in Parkinson's Disease Patients With Freezing of Gait: A Resting-State Functional Magnetic Resonance Imaging Study. Front Neurosci. 2020;14:582079.

8.	Sun H, Gan C, Cao X, Yuan Y, Zhang H, Wan C, et al. Microstructural and functional abnormalities of the locus coeruleus in freezing of gait in Parkinson's disease. Neurobiol Dis. 2025;208:106868.

9.	Wang S, Cai H, Cao Z, Li C, Wu T, Xu F, et al. More Than Just Static: Dynamic Functional Connectivity Changes of the Thalamic Nuclei to Cortex in Parkinson's Disease With Freezing of Gait. Front Neurol. 2021;12:735999.

10.	Zhou C, Zhong X, Yang Y, Yang W, Wang L, Zhang Y, et al. Alterations of regional homogeneity in freezing of gait in Parkinson's disease. J Neurol Sci. 2018;387:54–9.

11.	Wang M, Jiang S, Yuan Y, Zhang L, Ding J, Wang J, et al. Alterations of functional and structural connectivity of freezing of gait in Parkinson's disease. J Neurol. 2016;263(8):1583–92.

12.	Kou W, Wang X, Zheng Y, Zhao J, Cai H, Chen H, et al. Freezing of gait in Parkinson's disease is associated with the microstructural and functional changes of globus pallidus internus. Front Aging Neurosci. 2022;14:975068.

13.	Tessitore A, Amboni M, Esposito F, Russo A, Picillo M, Marcuccio L, et al. Resting-state brain connectivity in patients with Parkinson's disease and freezing of gait. Parkinsonism & Related Disorders. 2012;18(6):781–7.

14.	Mi TM, Mei SS, Liang PP, Gao LL, Li KC, Wu T, et al. Altered resting-state brain activity in Parkinson's disease patients with freezing of gait. Sci Rep. 2017;7(1):16711.

15.	Lenka A, Naduthota RM, Jha M, Panda R, Prajapati A, Jhunjhunwala K, et al. Freezing of gait in Parkinson's disease is associated with altered functional brain connectivity. Parkinsonism Relat Disord. 2016;24:100–6.

16.	Hu H, Chen J, Huang H, Zhou C, Zhang S, Liu X, et al. Common and specific altered amplitude of low-frequency fluctuations in Parkinson's disease patients with and without freezing of gait in different frequency bands. Brain Imaging Behav. 2020;14(3):857–68.

17.	Bharti K, Suppa A, Pietracupa S, Upadhyay N, Gianni C, Leodori G, et al. Aberrant functional connectivity in patients with Parkinson's disease and freezing of gait: a within- and between-network analysis. Brain Imaging Behav. 2020;14(5):1543–54.

18.	Wang S, Wu T, Li C, Wu T, Qian Y, Ren C, et al. Cerebral blood flow alterations specific to freezing of gait in Parkinson's disease. Neurol Sci. 2022;43(9):5323–31.

19.	Lench DH, DeVries W, Kearney-Ramos TE, Chesnutt A, Monsch ED, Embry AE, et al. Paired inhibitory stimulation and gait training modulates supplemental motor area connectivity in freezing of gait. Parkinsonism Relat Disord. 2021;88:28–33.

20.	Lench DH, Embry A, Malakouti N, DeTurk N, Revuelta GJ. Reduced automaticity in freezing of gait is associated with elevated cortico-cerebellar connectivity. Brain Imaging Behav. 2025;19(3):637–46.

21.	Steidel K, Ruppert MC, Palaghia I, Greuel A, Tahmasian M, Maier F, et al. Dopaminergic pathways and resting-state functional connectivity in Parkinson's disease with freezing of gait. Neuroimage Clin. 2021;32:102899.

22.	Droby A, Pelosin E, Putzolu M, Bommarito G, Marchese R, Mazzella L, et al. A Multimodal Imaging Approach Demonstrates Reduced Midbrain Functional Network Connectivity Is Associated With Freezing of Gait in Parkinson's Disease. Front Neurol. 2021;12:583593.

23.	Snijders AH, Leunissen I, Bakker M, Overeem S, Helmich RC, Bloem BR, et al. Gait-related cerebral alterations in patients with Parkinson’s disease with freezing of gait. Brain. 2011;134(1):59–72.

24.	Gilat M, Shine JM, Walton CC, O’Callaghan C, Hall JM, Lewis SJG. Brain activation underlying turning in Parkinson’s disease patients with and without freezing of gait: a virtual reality fMRI study. npj Parkinson's Disease. 2015;1(1):15020.

25.	Shine JM, Matar E, Ward PB, Bolitho SJ, Pearson M, Naismith SL, et al. Differential Neural Activation Patterns in Patients with Parkinson's Disease and Freezing of Gait in Response to Concurrent Cognitive and Motor Load. PLOS ONE. 2013;8(1):e52602.

26.	Xie H, Bai Y, Zhuang Y, Huang J, Wu D, Zhang X, et al. Theta-frequency subthalamic stimulation enhances conflict resolution in Parkinson's disease patients with freezing of gait through frontal cortex modulation. NPJ Parkinsons Dis. 2025;11(1):206.

27.	Camastra C, Augimeri A, Quattrone A, Quattrone A. Unveiling the neural network of freezing of gait in Parkinson's disease: A coordinate-based network study. J Parkinsons Dis. 2025;15(5):982–9.

28.	Rubino A, Assogna F, Piras F, Di Battista ME, Imperiale F, Chiapponi C, et al. Does a volume reduction of the parietal lobe contribute to freezing of gait in Parkinson's disease? Parkinsonism Relat Disord. 2014;20(10):1101–3.

29.	Jha M, Jhunjhunwala K, Sankara BB, Saini J, Kumar JK, Yadav R, et al. Neuropsychological and imaging profile of patients with Parkinson's disease and freezing of gait. Parkinsonism Relat Disord. 2015;21(10):1184–90.

30.	Tessitore A, Amboni M, Cirillo G, Corbo D, Picillo M, Russo A, et al. Regional gray matter atrophy in patients with Parkinson disease and freezing of gait. AJNR Am J Neuroradiol. 2012;33(9):1804–9.

31.	Vastik M, Hok P, Valosek J, Hlustik P, Mensikova K, Kanovsky P. Freezing of gait is associated with cortical thinning in mesial frontal cortex. Biomed Pap Med Fac Univ Palacky Olomouc Czech Repub. 2017;161(4):389–96.

32.	Kostić VS, Agosta F, Pievani M, Stefanova E, Ječmenica-Lukić M, Scarale A, et al. Pattern of brain tissue loss associated with freezing of gait in Parkinson disease. Neurology. 2012;78(6):409–16.

33.	Tard C, Delval A, Devos D, Lopes R, Lenfant P, Dujardin K, et al. Brain metabolic abnormalities during gait with freezing in Parkinson's disease. Neuroscience. 2015;307:281–301.

34.	Shine JM, Matar E, Ward PB, Bolitho SJ, Gilat M, Pearson M, et al. Exploring the cortical and subcortical functional magnetic resonance imaging changes associated with freezing in Parkinson's disease. Brain. 2013;136(Pt 4):1204–15.

35.	Shine JM, Matar E, Ward PB, Frank MJ, Moustafa AA, Pearson M, et al. Freezing of gait in Parkinson's disease is associated with functional decoupling between the cognitive control network and the basal ganglia. Brain. 2013;136(Pt 12):3671–81.

36.	Lench DH, Doolittle JD, Ramakrishnan V, Rowland N, Revuelta GJ. Subthalamic functional connectivity associated with freezing of gait dopa-response. Parkinsonism Relat Disord. 2024;118:105952.

37.	Matar E, Shine JM, Gilat M, Ehgoetz Martens KA, Ward PB, Frank MJ, et al. Identifying the neural correlates of doorway freezing in Parkinson's disease. Hum Brain Mapp. 2019;40(7):2055–64.

38.	Sarasso E, Agosta F, Piramide N, Canu E, Volonte MA, Filippi M. Brain activity of the emotional circuit in Parkinson's disease patients with freezing of gait. Neuroimage Clin. 2021;30:102649.

39.	Brugger F, Abela E, Hagele-Link S, Bohlhalter S, Galovic M, Kagi G. Do executive dysfunction and freezing of gait in Parkinson's disease share the same neuroanatomical correlates? J Neurol Sci. 2015;356(1-2):184–7.

40.	Hvingelby VS, Terkelsen MH, Johnsen EL, Moller M, Danielsen EH, Henriksen T, et al. Cholinergic patterns correlate with dopamine medication ON freezing of gait in Parkinson's disease. Brain. 2025;148(12):4508–18.

41.	Gan C, Ji M, Sun H, Cao X, Shi J, Wang L, et al. Dynamic functional connectivity reveals hyper-connected pattern and abnormal variability in freezing of gait of Parkinson's disease. Neurobiol Dis. 2023;185:106265.

42.	Potvin-Desrochers A, Atri A, Moreno AM, Paquette C. Levodopa alters resting-state functional connectivity more selectively in Parkinson's disease with freezing of gait. Eur J Neurosci. 2023;57(1):163–77.

43.	Fling BW, Cohen RG, Mancini M, Carpenter SD, Fair DA, Nutt JG, et al. Functional Reorganization of the Locomotor Network in Parkinson Patients with Freezing of Gait. PLOS ONE. 2014;9(6):e100291.

44.	Sunwoo MK, Cho KH, Hong JY, Lee JE, Sohn YH, Lee PH. Thalamic volume and related visual recognition are associated with freezing of gait in non-demented patients with Parkinson's disease. Parkinsonism Relat Disord. 2013;19(12):1106–9.

45.	Pietracupa S, Suppa A, Upadhyay N, Gianni C, Grillea G, Leodori G, et al. Freezing of gait in Parkinson's disease: gray and white matter abnormalities. J Neurol. 2018;265(1):52–62.

46.	Guo M, Liu H, Gao L, Yu H, Ren Y, Li Y, et al. A radiomics approach for predicting gait freezing in Parkinson's disease based on resting-state functional magnetic resonance imaging indices: A cross-sectional study. Neural Regen Res. 2026;21(4):1621–7.

47.	Herman T, Rosenberg-Katz K, Jacob Y, Giladi N, Hausdorff JM. Gray matter atrophy and freezing of gait in Parkinson's disease: Is the evidence black-on-white? Mov Disord. 2014;29(1):134–9.

48.	Peterson DS, Smulders K, Mancini M, Nutt JG, Horak FB, Fling BW. Relating Response Inhibition, Brain Connectivity, and Freezing of Gait in People with Parkinson's Disease. J Int Neuropsychol Soc. 2021;27(7):733–43.

49.	Jin C, Qi S, Yang L, Teng Y, Li C, Yao Y, et al. Abnormal functional connectivity density involvement in freezing of gait and its application for subtyping Parkinson's disease. Brain Imaging Behav. 2023;17(4):375–85.

50.	Jin C, Yang L, Qi S, Teng Y, Li C, Yao Y, et al. Structural Brain Network Abnormalities in Parkinson's Disease With Freezing of Gait. Front Aging Neurosci. 2022;14:944925.

51.	de Lima-Pardini AC, Coelho DB, Nucci MP, Boffino CC, Batista AX, de Azevedo Neto RM, et al. Brain networks associated with anticipatory postural adjustments in Parkinson's disease patients with freezing of gait. Neuroimage Clin. 2020;28:102461.

52.	Moreira-Neto A, Ugrinowitsch C, Coelho DB, de Lima-Pardini AC, Barbosa ER, Teixeira LA, et al. Freezing of gait, gait initiation, and gait automaticity share a similar neural substrate in Parkinson's disease. Hum Mov Sci. 2022;86:103018.

53.	Huang HC, Chen CM, Lu MK, Liu BL, Li CI, Chen JC, et al. Gait-Related Brain Activation During Motor Imagery of Complex and Simple Ambulation in Parkinson's Disease With Freezing of Gait. Front Aging Neurosci. 2021;13:731332.

54.	Silva-Batista C, Lira J, Coelho DB, de Lima-Pardini AC, Nucci MP, Mattos ECT, et al. Mesencephalic Locomotor Region and Presynaptic Inhibition during Anticipatory Postural Adjustments in People with Parkinson's Disease. Brain Sci. 2024;14(2).

55.	Youn J, Lee JM, Kwon H, Kim JS, Son TO, Cho JW. Alterations of mean diffusivity of pedunculopontine nucleus pathway in Parkinson's disease patients with freezing of gait. Parkinsonism Relat Disord. 2015;21(1):12–7.

56.	Li E, Ruan X, Li Y, Zhang G, Li M, Wei X. Exploring Cortical Thickness Alteration in Parkinson Disease Patients with Freezing of Gaits. Neural Plast. 2020;2020:8874119.

57.	Peterson DS, Fling BW, Mancini M, Cohen RG, Nutt JG, Horak FB. Dual-task interference and brain structural connectivity in people with Parkinson's disease who freeze. J Neurol Neurosurg Psychiatry. 2015;86(7):786–92.

58.	Wang L, Gan C, Sun H, Ji M, Zhang H, Cao X, et al. Impaired structural and reserved functional topological organizations of brain networks in Parkinson's disease with freezing of gait. Quant Imaging Med Surg. 2023;13(1):66–79.

59.	Maidan I, Jacob Y, Giladi N, Hausdorff JM, Mirelman A. Altered organization of the dorsal attention network is associated with freezing of gait in Parkinson's disease. Parkinsonism Relat Disord. 2019;63:77–82.

60.	Myers PS, McNeely ME, Koller JM, Earhart GM, Campbell MC. Cerebellar Volume and Executive Function in Parkinson Disease with and without Freezing of Gait. J Parkinsons Dis. 2017;7(1):149–57.

61.	Gilat M, Ehgoetz Martens KA, Miranda-Domínguez O, Arpan I, Shine JM, Mancini M, et al. Dysfunctional Limbic Circuitry Underlying Freezing of Gait in Parkinson’s Disease. Neuroscience. 2018;374:119–32.

62.	D'Cruz N, Vervoort G, Chalavi S, Dijkstra BW, Gilat M, Nieuwboer A. Thalamic morphology predicts the onset of freezing of gait in Parkinson's disease. NPJ Parkinsons Dis. 2021;7(1):20.

63.	Onder H, Oguz KK, Has AC, Elibol B. Comparative analysis of freezing of gait in distinct Parkinsonism types by diffusion tensor imaging method and cognitive profiles. J Neural Transm (Vienna). 2023;130(4):521–35.

64.	Wrobel PP, Peter A, Kirsten M, Gulberti A, Bester M, Goebell E, et al. Supplementary motor area microstructure defines the extent of gait impairment in Parkinson's disease. NPJ Parkinsons Dis. 2025;11(1):260.

65.	Ehgoetz Martens KA, Hall JM, Georgiades MJ, Gilat M, Walton CC, Matar E, et al. The functional network signature of heterogeneity in freezing of gait. Brain. 2018;141(4):1145–60.

66.	van der Hoorn A, Renken RJ, Leenders KL, de Jong BM. Parkinson-related changes of activation in visuomotor brain regions during perceived forward self-motion. PLoS One. 2014;9(4):e95861.

67.	Potvin-Desrochers A, Mitchell T, Gisiger T, Paquette C. Changes in Resting-State Functional Connectivity Related to Freezing of Gait in Parkinson's Disease. Neuroscience. 2019;418:311–7.

68.	Gallea C, Wicki B, Ewenczyk C, Rivaud-Pechoux S, Yahia-Cherif L, Pouget P, et al. Antisaccade, a predictive marker for freezing of gait in Parkinson's disease and gait/gaze network connectivity. Brain. 2021;144(2):504–14.

69.	Li N, Lei D, Peng J, Suo X, Li J, Duan L, et al. Brain network topology and future development of freezing of gait in Parkinson’s disease: a longitudinal study. Journal of Neurology. 2022;269(5):2503–12.

70.	Peterson DS, Pickett KA, Duncan R, Perlmutter J, Earhart GM. Gait-related brain activity in people with Parkinson disease with freezing of gait. PLoS One. 2014;9(3):e90634.

71.	Ruan X, Li Y, Li E, Xie F, Zhang G, Luo Z, et al. Impaired Topographical Organization of Functional Brain Networks in Parkinson's Disease Patients With Freezing of Gait. Front Aging Neurosci. 2020;12:580564.

72.	Li N, Suo X, Zhang J, Lei D, Wang L, Li J, et al. Disrupted functional brain network topology in Parkinson's disease patients with freezing of gait. Neurosci Lett. 2021;759:135970.

73.	Zhou W, He J, Zhang C, Pan Y, Sang T, Qiu X. Fiber-specific white matter alterations in Parkinson's disease patients with freezing of gait. Brain Res. 2023;1815:148440.

74.	Zhang Y, Zhang C, Wang X, Liu Y, Jin Z, Haacke EM, et al. Iron and neuromelanin imaging in basal ganglia circuitry in Parkinson's disease with freezing of gait. Magn Reson Imaging. 2024;111:229–36.

75.	Fling BW, Cohen RG, Mancini M, Nutt JG, Fair DA, Horak FB. Asymmetric pedunculopontine network connectivity in parkinsonian patients with freezing of gait. Brain. 2013;136(8):2405–18.

76.	Gan C, Cao X, Wang L, Sun H, Ji M, Zhang H, et al. Cholinergic basal forebrain atrophy in Parkinson's disease with freezing of gait. Ann Clin Transl Neurol. 2023;10(5):814–24.

77.	Yang Y, Zhang M, Yan Y, Chang Y, Chen M. Relationship between freezing of gait in Parkinson disease and locus coeruleus dysfunction: a neuromelanin-sensitive magnetic resonance imaging study. Quant Imaging Med Surg. 2025;15(1):911–20.

78.	Gan L, Yan R, Su D, Liu Z, Miao G, Wang Z, et al. Alterations of structure and functional connectivity of visual brain network in patients with freezing of gait in Parkinson's disease. Front Aging Neurosci. 2022;14:978976.

79.	Bharti K, Suppa A, Pietracupa S, Upadhyay N, Gianni C, Leodori G, et al. Abnormal Cerebellar Connectivity Patterns in Patients with Parkinson's Disease and Freezing of Gait. Cerebellum. 2019;18(3):298–308.

80.	Quek DYL, Taylor N, Gilat M, Lewis SJG, Ehgoetz Martens KA. Effect of dopamine on limbic network connectivity at rest in Parkinson's disease patients with freezing of gait. Transl Neurosci. 2024;15(1):20220336.

81.	Lv L, Zhang H, Tan X, Long Z, Qin L, Bai R, et al. Associated factors and abnormal dorsal raphe nucleus connectivity patterns of freezing of gait in Parkinson's disease. J Neurol. 2022;269(12):6452–66.

82.	Vervoort G, Heremans E, Bengevoord A, Strouwen C, Nackaerts E, Vandenberghe W, et al. Dual-task-related neural connectivity changes in patients with Parkinson' disease. Neuroscience. 2016;317:36–46.

83.	Jin C, Qi S, Teng Y, Li C, Yao Y, Ruan X, et al. Altered Degree Centrality of Brain Networks in Parkinson's Disease With Freezing of Gait: A Resting-State Functional MRI Study. Front Neurol. 2021;12:743135.

84.	Huang LC, Chen LG, Kao HW, Lin SH, Tsai ST, Wu PA, et al. Functional connectivity compensation in Parkinson's disease with freezing of gait. Eur J Neurosci. 2024;60(9):6279–90.

85.	Canu E, Agosta F, Sarasso E, Volonte MA, Basaia S, Stojkovic T, et al. Brain structural and functional connectivity in Parkinson's disease with freezing of gait. Hum Brain Mapp. 2015;36(12):5064–78.

86.	Vastik M, Hok P, Hlustik P, Otruba P, Tüdös Z, Kanovsky P. Botulinum toxin treatment of freezing of gait in Parkinson's disease patients as reflected in functional magnetic resonance imaging of leg movement. Neuro Endocrinol Lett. 2016;37(2):147–53.

87.	Sacmaci H, Erkoc MF, Akturk T. Measurement of the facial nerve thickness and its correlation with freezing phenomenon and hypomimia in Parkinson's disease. Clin Neurol Neurosurg. 2021;210:106960.

88.	Sarasso E, Basaia S, Cividini C, Stojkovic T, Stankovic I, Piramide N, et al. MRI biomarkers of freezing of gait development in Parkinson’s disease. npj Parkinson's Disease. 2022;8(1):158.

89.	Youn J, Cho JW, Lee WY, Kim GM, Kim ST, Kim HT. Diffusion tensor imaging of freezing of gait in patients with white matter changes. Mov Disord. 2012;27(6):760–4.

90.	Trufanov AG, Khalimov RR, Yurin AA, Litvinenko IV. Use of Magnetic Resonance Tractography in Detecting Specific Features of the Impairment of Conduction Pathways and Their Prognostic Value in Parkinson’s Disease Complicated by Freezing of Gait. Sovremennye tehnologii v medicine. 2016;8(4):178–84.

91.	Tard C, Delval A, Defebvre L, Lenfant P, Devos D, Moreau C, et al. Metabolic connectivity of freezing in Parkinson's disease. J Parkinsons Dis. 2025;15(1):154–62.

92.	Zhou Y, Zhao J, Hou Y, Su Y, Chan P, Wang Y. Dopaminergic pathway and primary visual cortex are involved in the freezing of gait in Parkinson's disease: a PET-CT study. Neuropsychiatr Dis Treat. 2019;15:1905–14.

93.	Bartels AL, de Jong BM, Giladi N, Schaafsma JD, Maguire RP, Veenma L, et al. Striatal dopa and glucose metabolism in PD patients with freezing of gait. Mov Disord. 2006;21(9):1326–32.

94.	Mitchell T, Potvin-Desrochers A, Lafontaine AL, Monchi O, Thiel A, Paquette C. Cerebral Metabolic Changes Related to Freezing of Gait in Parkinson Disease. J Nucl Med. 2019;60(5):671–6.

95.	McKay JL, Nye J, Goldstein FC, Sommerfeld B, Smith Y, Weinshenker D, et al. Levodopa responsive freezing of gait is associated with reduced norepinephrine transporter binding in Parkinson's disease. Neurobiol Dis. 2023;179:106048.

96.	Imamura K, Okayasu N, Nagatsu T. Cerebral blood flow and freezing of gait in Parkinson's disease. Acta Neurol Scand. 2012;126(3):210–8.

97.	Matsui H, Udaka F, Miyoshi T, Hara N, Tamaura A, Oda M, et al. Three-dimensional stereotactic surface projection study of freezing of gait and brain perfusion image in Parkinson's disease. Mov Disord. 2005;20(10):1272–7.

98.	Miranda-Dominguez O, Ragothaman A, Hermosillo R, Feczko E, Morris R, Carlson-Kuhta P, et al. Lateralized Connectivity between Globus Pallidus and Motor Cortex is Associated with Freezing of Gait in Parkinson's Disease. Neuroscience. 2020;443:44–58.

99.	Wang X, Chen H, Ma X, Liu H, Wu D, Du W, et al. Atrophy of ventral diencephalon is associated with freezing of gait in Parkinson's disease: analysis of two cohorts. NPJ Parkinsons Dis. 2025;11(1):44.

100.	Lench DH, Keith K, Wilson S, Padgett L, Benitez A, Ramakrishnan V, et al. Neurodegeneration of the Globus Pallidus Internus as a Neural Correlate to Dopa-Response in Freezing of Gait. J Parkinsons Dis. 2022;12(4):1241–50.

101.	Naduthota RM, Honnedevasthana AA, Lenka A, Saini J, Geethanath S, Bharath RD, et al. Association of freezing of gait with nigral iron accumulation in patients with Parkinson's disease. J Neurol Sci. 2017;382:61–5.

102.	Roytman S, Paalanen R, Griggs A, David S, Pongmala C, Koeppe RA, et al. Cholinergic system correlates of postural control changes in Parkinson's disease freezers. Brain. 2023;146(8):3243–57.

103.	Taylor NL, Wainstein G, Quek D, Lewis SJG, Shine JM, Ehgoetz Martens KA. The Contribution of Noradrenergic Activity to Anxiety-Induced Freezing of Gait. Movement Disorders. 2022;37(7):1432–43.

104.	Maillet A, Thobois S, Fraix V, Redoute J, Le Bars D, Lavenne F, et al. Neural substrates of levodopa-responsive gait disorders and freezing in advanced Parkinson's disease: a kinesthetic imagery approach. Hum Brain Mapp. 2015;36(3):959–80.

105.	Chou KL, Kanel P, van Emde Boas M, Roytman S, Carli G, Albin RL, et al. Cholinergic System Changes in Dopa-Unresponsive Freezing of Gait in Parkinson's Disease. Mov Disord. 2025;40(8):1584–94.

106.	Raghu ALB, Parker T, Zand APD, Payne S, Andersson J, Stein J, et al. Tractography patterns of pedunculopontine nucleus deep brain stimulation. J Neural Transm (Vienna). 2021;128(5):659–70.

107.	Hall JM, Shine JM, Ehgoetz Martens KA, Gilat M, Broadhouse KM, Szeto JYY, et al. Alterations in white matter network topology contribute to freezing of gait in Parkinson's disease. J Neurol. 2018;265(6):1353–64.

108.	Vercruysse S, Leunissen I, Vervoort G, Vandenberghe W, Swinnen S, Nieuwboer A. Microstructural changes in white matter associated with freezing of gait in Parkinson's disease. Mov Disord. 2015;30(4):567–76.
